# Radiologic Insights: Diagnosing Lumbosacral Transitional Vertebrae. Systematic Review of the Literature

**DOI:** 10.1111/papr.70138

**Published:** 2026-03-09

**Authors:** Pedro Andrade‐Andrade, Juan Carlos Acevedo‐González

**Affiliations:** ^1^ Facultad de Medicina, Universidad de Los Andes Bogotá Colombia; ^2^ Departamento de Neurociencias, Facultad de Medicina Pontificia Universidad Javeriana, Hospital Universitario San Ignacio Bogotá Colombia

**Keywords:** Castellvi classification, low back pain, lumbosacral transitional vertebra, radiology, spine, systematic review

## Abstract

**Introduction:**

The lumbosacral transitional vertebra (LSTV) has been studied since 1876, with Castellvi developing a classification in 1984 based on its anatomy and laterality. It often goes unnoticed, or its diagnosis is limited to a lumbar spine X‐ray for confirmation. This has led to LSTV being underdiagnosed or even ignored. Our aim is to describe and evaluate radiological diagnostic techniques for LSTV and propose a diagnostic methodology to reduce errors in vertebral level identification, useful for percutaneous procedures and/or biomechanical measurement analysis.

**Materials and Methods:**

A systematic literature review was conducted. The search terms included: “Castellvi,” “Lumbosacral Transitional Vertebra,” “Radiology.” Logical connectors such as “and” and “or” were applied. The following databases were reviewed: Scopus, PubMed, Ovid, ScienceDirect, EBSCO, and Nature. The timeframe was limited from 2004 to December 2024. Inclusion and exclusion criteria were applied. A total of 419 articles were identified. The “Rayyan” program was used to compile information, and “PRISMA,” “STROBE,” and “CONSORT” were used to facilitate the analysis process.

**Results:**

Forty‐eight articles were included and analyzed (10 CT, 4 PET‐CT, 2 bone scans, 9 MRI, 6 X‐rays, 4 EOS, and 13 mixed). The most common findings highlighted CT as the gold standard for diagnosing LSTV, with spinopelvic parameters correlating with LSTV. Radiography is effective for vertebral numbering. MRI studies utilize anatomical landmarks to identify vertebral levels and LSTV, although they are less sensitive. EOS is also used for vertebral level identification.

**Conclusions:**

Our proposed diagnostic methodology for LSTV includes: first, using plain AP radiography for cranial‐to‐caudal vertebral numbering and evaluating morphological anomalies. Second, if LSTV is suspected, performing CT as the gold standard for diagnosis due to its high sensitivity and specificity, and measuring spinopelvic parameters to correlate with LSTV. Third, using MRI in special cases. Fourth, conducting a morphological analysis and using Jenkins' classification for LSTV categorization.

**Abbreviations**
AP: anteroposterior
CT: computed tomography
LBP: low back pain
LSTV: lumbosacral transitional vertebra
MRI: magnetic resonance imaging
PET‐CT: positron emission tomography
PI: pelvic incidence
SPECT: single‐photon emission computed tomography

## 1 Introduction

The clinical relevance of Lumbosacral Transitional Vertebra (LSTV) has been a subject of prolonged debate. In 1876, Rosenberg proposed that the spine underwent a shortening process as a mechanism to stabilize the erect column. He suggested that the sacralization of the fifth lumbar vertebra represented an anthropological progression, while the lumbarization of the first sacral vertebra was considered regressive [1]. Subsequently, Bertolotti et al. [2] explored in 1917 the association between low back pain (LBP) and the extension of the fifth lumbar vertebra to the sacrum, giving rise to Bertolotti's syndrome. Later, in 1955, Stinchfield and Sinton [1] conducted a radiological study and referenced herniations at the level of the transitional vertebra.

**FIGURE 1:**
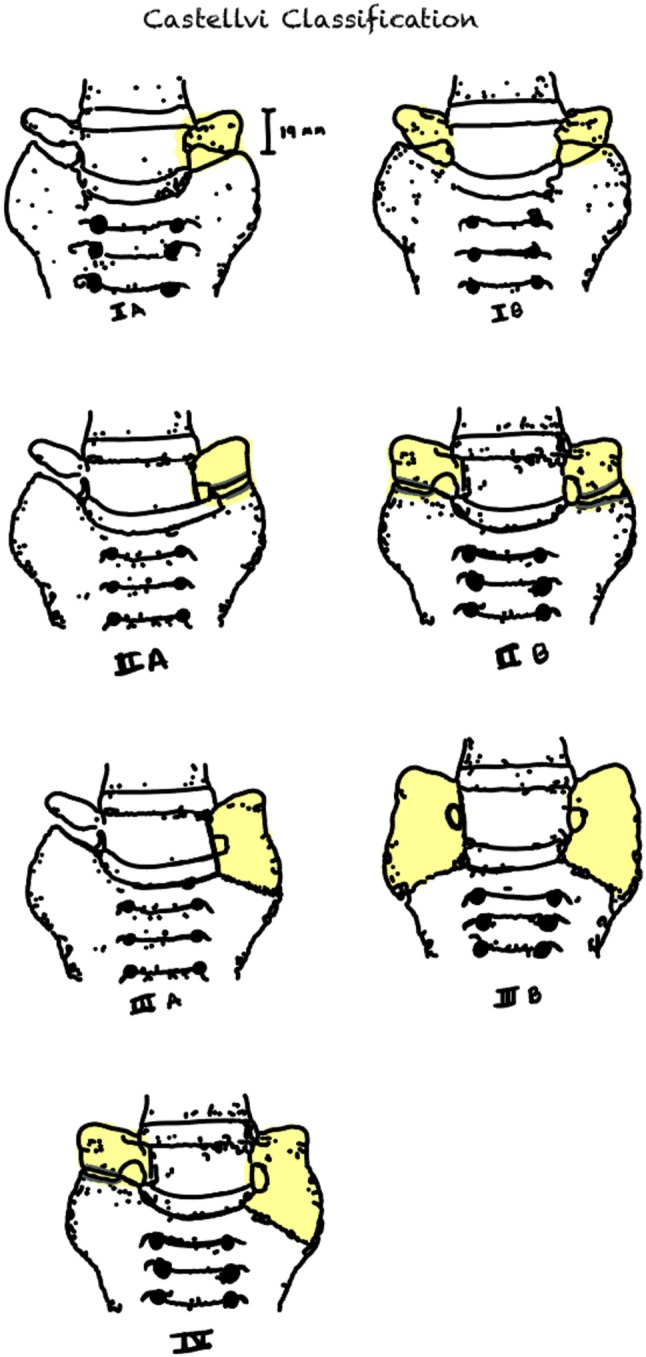
Graphic illustration of Castellvi classification. Four categories with seven subcategories.

Years later, in 1984, Castellvi et al. [3] conducted a retrospective study including 200 consecutive cases from 1979 with myelographic evidence of herniated nucleus pulposus. The myelograms were initially interpreted by neuroradiology personnel and later independently reviewed by one of the authors. On the basis of this, they developed the Castellvi classification of LSTV into four categories with seven subcategories (Figure 1) according to the morphological characteristics in radiology and the clinical relevance concerning lumbar herniation [3].

**Type I**. *Dysplastic transverse process*: a, unilateral; b, bilateral.

This type is characterized by a large, triangular transverse process measuring at least 19 mm in width, as originally described by Southworth and Bersack in 1950 [3].

**Type II**. *Incomplete lumbarization/sacralization*: a, unilateral; b, bilateral.

This type is characterized by a prominent transverse process that appears to fit the shape of the sacral ala. They are considered incomplete due to the presence of a diarthrodial joint between the transverse process and the sacrum [3].

**Type III**. *Complete lumbarization/sacralization*: a, unilateral; b, bilateral.

Similar to type II, but distinguished by a true osseous union between the transverse process and the sacrum instead of a diarthrodial joint [3].

**Type IV**. *Mixed*: Patients in this category exhibit Type II on one side and Type III on the other.

The terms “lumbarization” and “sacralization” are used because the exact number of vertebrae in the patients' spinal column could not be definitively determined [3].

Given the historical context, the enlargement of the transverse process on one or both sides of the last lumbar vertebra, or the formation of a joint or complete fusion between this process and the sacral ala, constitutes an anomaly that has been and remains common. This anomaly often leads to what is known as LSTV, in which the last lumbar segment may present sacralization, or the first sacral segment may present lumbarization. Occasionally, a spectrum of morphological changes in these vertebral bodies can be observed, described as “squared” and “wedging” [4, 5].

LSTV is a clinically relevant anatomical alteration due to its association with LBP, known as Bertolotti syndrome, which may be of primary origin, caused by intrinsic inflammatory processes, or secondary, resulting from biomechanical alterations in the spine. Its identification is crucial for planning surgical or percutaneous procedures, especially in minimally invasive techniques such as spinal fusions or epidural injections, and for adjusting sagittal balance analysis protocols, which are designed under the premise of five lumbar vertebrae. This number can vary in the presence of LSTV or other anatomical anomalies. Nonetheless, standardizing radiological findings to correctly identify and classify this condition according to Castellvi's criteria remains a challenge, highlighting the need for more consistent and precise diagnostic approaches.

The aim of this systematic review is to describe and evaluate radiological diagnostic techniques for LSTV and propose a diagnostic methodology to reduce errors in identifying vertebral levels, useful for percutaneous procedures and/or biomechanical measurement analysis.

## 2 Materials and Methods

### 2.1 Literature Review

A systematic review of medical literature from the last 21 years was conducted. The following search terms were used in databases: “Castellvi,” “Lumbosacral Transitional Vertebra,” and “Radiology,” using logical connectors such as “AND” and “OR.” Databases like Scopus, PubMed, Ovid, ScienceDirect, EBSCO, and Nature were reviewed. The search period was limited from 2004 to December 2024. The selected articles were organized using the systematic review tool “Rayyan.” This application is a platform that allows easy manipulation of the collected information.

### 2.2 Article Selection

A total of 419 articles were identified from the databases: Scopus (*n* = 149), PubMed (*n* = 166), Ovid (*n* = 54), ScienceDirect (*n* = 35), EBSCO (*n* = 11), and Nature (*n* = 4). Each author independently reviewed the abstract of each article found and applied the following inclusion and exclusion criteria.

The *inclusion criteria* were:

1. Studies in English or Spanish that included a radiological description of LSTV, either findings or diagnostic technique used.
2. Articles conducted on humans.
3. Articles published between January 1, 2004, and December 18, 2024.

The *exclusion criteria* were:

1. Articles not conducted on humans.
2. Cadaveric studies.
3. Technical notes or editor's notes.

**FIGURE 2:**
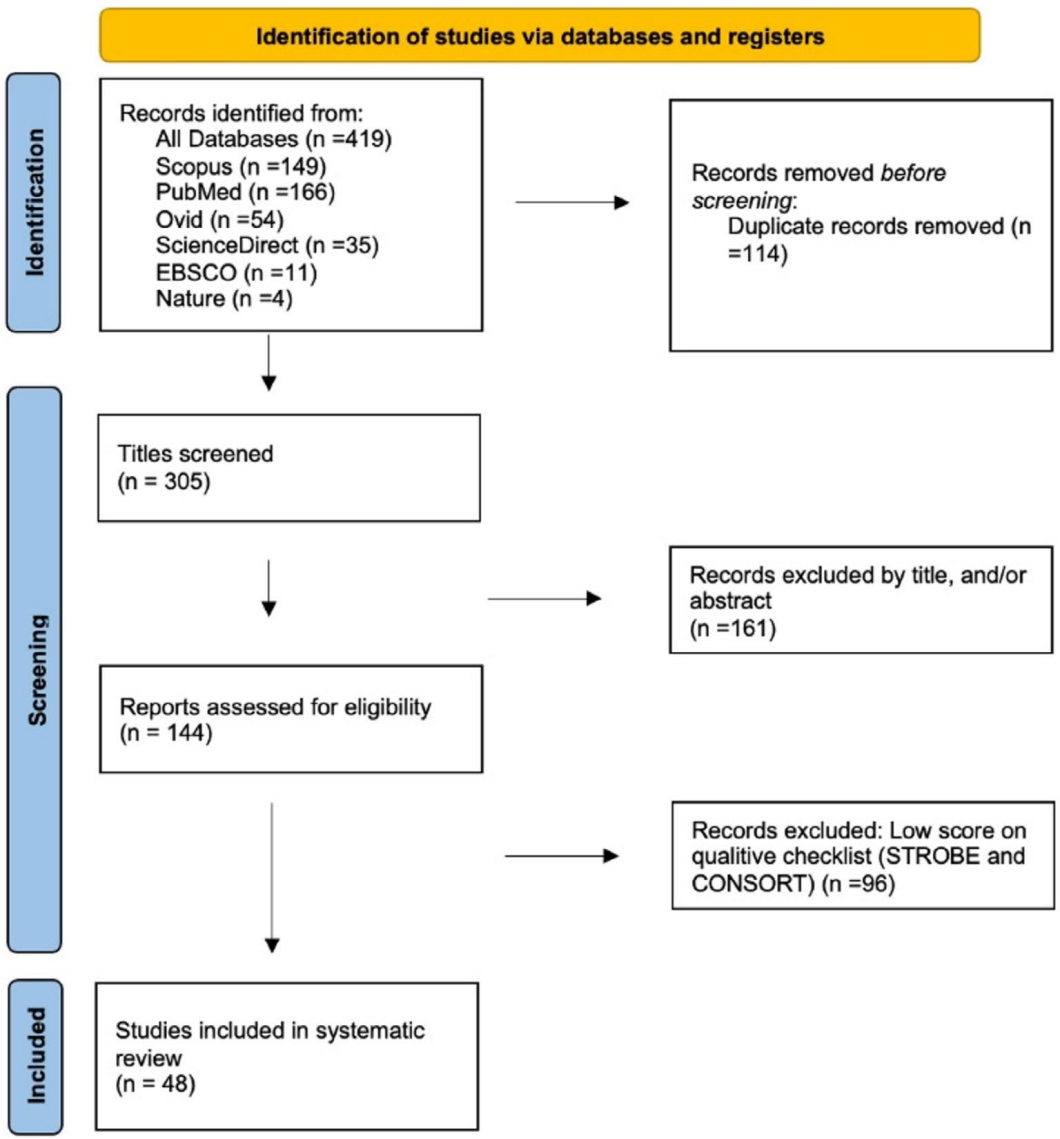
Preferred Reporting Items for Systematic reviews and Meta‐Analyses (PRISMA) diagram. CONSORT, Consolidated Standards of Reporting Trials; STROBE, Strengthening the Reporting of Observational Studies in Epidemiology.

A thorough evaluation of all selected articles was performed using specific qualitative checklists for each study type: Strengthening the Reporting of Observational Studies in Epidemiology (STROBE) for observational studies [6] and Consolidated Standards of Reporting Trials (CONSORT) for randomized clinical trials [7]. All articles included in this analysis are original, ensuring the absence of duplication in the reviewed literature. The systematic review was conducted following the structure and recommendations outlined in the Preferred Reporting Items for Systematic Reviews and Meta‐Analyses (PRISMA) guidelines [8]. Additionally, the PRISMA diagram was incorporated to visually represent the article selection process (Figure 2).

Finally, 48 articles were included. Among these, there was 1 case series study [9], 2 case reports [10, 11], 2 literature reviews [4, 12], 4 prospective studies [5, 13, 14, 15], 10 cross‐sectional studies [16, 17, 18, 19, 20, 21, 22, 23, 24, 25], and 29 retrospective studies [26, 27, 28, 29, 30, 31, 32, 33, 34, 35, 36, 37, 38, 39, 40, 41, 42, 43, 44, 45, 46, 47, 48, 49, 50, 51, 52, 53, 54].

## 3 Results

A total of 48 articles were included in the systematic review. A summary of the included studies is shown in Table 1. The results of the selected articles are categorized according to the diagnostic techniques used for the diagnosis of LSTV, such as CT, PET‐CT, scintigraphy, MRI, radiography, EOS, and the combined use of these techniques.

**TABLE 1:** Summary of the systematic review. Title of the article, first author and year, methodology used by the authors, study design, aim, conclusions, and radiologic image used.

Title	First author and year	Methodology	Design	Aim	Conclusions	Radiologic image used
18F‐sodium fluoride bone PET‐CT in symptomatic lumbosacral transitional vertebra	Usmani 2020 [9]	The study population comprised 55 patients with LSTV. All patients underwent integrated positron‐emission tomography (PET)/computed tomography (CT) with 18F‐NaF. A three‐point grading system was used to evaluate 18F‐NaF uptake and the results were analyzed	Case Series	To analyze the relationship between 18F‐labeled sodium fluoride (NaF) uptake and lumbar back pain in patients with LSTV	18F‐NaF PET/CT can be useful in evaluating back pain and 18F‐NaF may be used as an adjunctive biological maker for assessing LSTV as a potential cause of pain	PET‐CT
Bertolotti syndrome demonstrated on 18F‐NaF PET/CT	Usmani 2017 [10]	Case report	Case report	Case	18F‐NaF PET/CT can be useful in evaluation of some uncommon causes of low‐back pain. Bertolotti syndrome should always be included in the differential diagnosis of lower‐back pain in young patients	PET‐CT
Lumbosacral transitional vertebra diagnosed on 99mTc‐methylene diphosphonate SPECT/CT	Kassir 2015 [11]	Case report	Case report	Case	This case shows that a whole‐body bone scan with hybrid SPECT/CT imaging of the area of pain can be a tool to help diagnose LSTV	Scintigraphy
A review of lumbosacral transitional vertebrae and associated vertebral numeration	Lian 2018 [12]	A PubMed and EMBASE search using various combinations of specific key words including “LSTV,” “lumbosacral transitional vertebrae,” “count,” “vertebral numbering,” and “number” was performed	Literature review	To review the current literature on methods of accurate numeration of vertebral segments in patients with Lumbosacral transitional vertebrae (LSTVs)	LSTV and spinal numerical variants are common. When MRI of the lumbar spine is indicated, physicians should consider an additional scout. Whole spine imaging remains the most accurate method for vertebral numeration in the setting of transitional vertebrae, and may identify otherwise undiagnosed cervical or thoracic anomalies	Mixed
Lumbosacral transitional vertebrae: classification, imaging findings, and clinical relevance	Konin 2010 [4]	Literature review	Literature review	Address each of the problems with attention to correctly identifying and numbering the LSTV, as well as detecting imaging findings related to the genesis of low back pain	LSTVs are common anomalies of the spine necessitating the ability to accurately identify and number the affected segment. Although it has been long contested, there is convincing evidence of an association of low back pain with LSTV. Knowledge of the biomechanical alterations within the spine caused by LSTVs will aid the radiologist in understanding and recognizing the imaging findings seen in patients with low back pain and a transitional segment.	Mixed
Bertolotti's syndrome in low‐backache population: Classification and imaging findings	Ravikanth 2019 [5]	Five hundred lumbosacral radiographs of LBP patients were examined after obtaining prior consent from the patient and approval from the “institutional ethics committee.” Data collection consisted of the patient's age at the time of imaging gender and number of lumbar vertebral bodies. 134 were classified as positive for sacralization. Dysplastic transverse processes were classified according to the Castellvi radiographic classification system. The incidence of sacralization in patients and the control groups was reported, and the anomaly was compared according to the groups	Prospective study	To classify the anatomical variations in LSTV and determine, by plain radiography, if there exists a relationship between sacralization and low backache (LBP)	Accurate enumeration of LSTV and communication to the referring clinician will help avoid such dreaded complications as wrong‐level spine surgery. Lumbosacral transitional segments are a common cause in the low‐backache population. In comparison with the nonspecific low‐backache group, the VAS scores were significantly higher and the pain duration was significantly longer in the LSTV group	Radiographs
Effect of sacralization on the success of lumbar transforaminal epidural steroid injection treatment: Prospective clinical trial	Sencan 2023 [13]	The study included 64 patients diagnosed with radicular low back pain due to unilateral and single‐level lumbar disk herniation. Patients were divided into two groups: patients with sacralization (Group S) and patients without lumbosacral transitional vertebrae (Group A). Injection was applied to the relevant level. Patients were evaluated with Numeric Rating Scale and Modified Oswestry Disability Index before, at week 3 and month 3 after the procedure. Sacralization presence was determined by MRI. Sacralization was categorized by anteroposterior lumbar radiography using Castellvi classification. Treatment success was considered as ≥ 50% reduction in NRS scores	Prospective study	To investigate the effect of the sacralization on the results of transforaminal epidural steroid injection for radicular low back pain	Transforaminal epidural steroid injection is an effective and safe method for radicular low back pain. Sacralization presence should be evaluated before treatment considering that it may be a risk factor reducing treatment success	Mixed
Lumbar sacralization and L4–L5 microdiscectomy, a prospective cohort study on radiologic and clinical outcomes	Omidi 2024 [14]	This prospective cohort study was conducted in a university referral hospital. The patients with L4–L5 disc herniation and eligible for microdiscectomy were enrolled and allocated in G1 (with LS) and G2 (no LS). After the L4–L5 microdiscectomy patients were followed, clinical and radiological parameters were collected to investigate the influence on the outcomes. Recurrence, low back outcome score (LBOS), and the Oswestry disability index (ODI) were defined as main outcomes	Prospective study	To evaluate the role of lumbar sacralization (LS) on the surgical outcomes of L4–L5 microdiscectomy	L4–L5 microdiscectomy in patients with lumbar sacralization was associated with higher recurrence rates, worse ODI and LBOS scores, persistent postoperative axial back pain, and radicular pain	Mixed
Normative spino‐pelvic parameters in patients with the lumbarization of S1 compared to a normal asymptomatic population	Price 2016 [15]	Two databases of asymptomatic patients were combined to identify 11 patients with the lumbarization of S1. The whole spine images were used to measure the true prevalence rate. Lumbar 3D EOS models were built to measure spino‐pelvic parameters for the lumbarization group compared to the asymptomatic population. Seven patients appeared at first to have six lumbar vertebrae, but counting caudally from C2 showed this was not the case	Prospective study	To give normative values for PI, SS, pelvic tilt (PT), and total lumbar lordosis in the lumbarization of S1 and to investigate the correlations between lumbar lordosis with PI, as measured at the first non‐mobile sacral vertebrae, S2, and L6 incidence (L6I), at the true S1	Incomplete imaging of the spine may lead to false estimation of the prevalence of lumbarization. Patients with lumbarization have higher lordosis values and lordosis can now be estimated during pre‐operative planning for this group	EOS
A whole‐spine magnetic resonance imaging‐based cross‐sectional study of the clinicoradiological association of lumbosacral transitional vertebra with degenerative disc disease, end plate degeneration, low back pain, and facet tropism	Bhagchandani 2024 [25]	Whole‐spine magnetic resonance imaging was evaluated for disc degeneration using Pfirrmann grading, end plate changes using total end plate score (TEPS), and facet tropism in patients with low back pain (LBP) and radicular pain (RP), and their association with LSTV was analyzed	Cross‐sectional study	To understand lumbosacral transitional vertebra (LSTV)–associated degenerative pathologies and their correlation to low back pain and radicular pain	Patients with sacralization accounted for > 80% of patients with LSTV with either LBP or radiculopathy, which may be explained by the high incidence of disc degeneration of all three levels immediately proximal to a sacralized vertebra as well as a high incidence of end plate degeneration and facet tropism	MRI
Diagnostic limitations and aspects of the lumbosacral transitional vertebrae (LSTV)	Landauer 2022 [16]	In the scoliosis outpatient clinic, 1482 patients were radiologically examined, and ambiguous lumbosacral junction underwent MRI examination. 115 patients with Castellvi classification type II–IV were included and the results were compared with the literature in PubMed (October 12, 2022). Patients with Castellvi I classification (height of L5 transverse process > 19 mm) were not included in the study. This decision was made in the initial phase of the study, because in their unpublished studies the imaging inaccuracy allows an exact measurement of the L5 transverse process only in the Ferguson image. However, this additional X‐ray is not permitted for reasons of radiation hygiene. Only patients with Castellvi classification type II–IV were included in the study	Cross‐sectional study	The lumbosacral transitional vertebrae (LSTV) offer itself as a model for IVD (intervertebral disc) regeneration. The aim of this work is to support this statement	Only the Ferguson image (30° angle AP radiograph) serves as a standard method for detecting LSTV. Additional imaging is prohibited for radiation hygiene reasons. The alternative is an MRI examination, which provides additional information in all three planes in space. The standard images for disc assessment (coronal and sagittal) are not sufficient for the assessment of LSTV. In conclusion, the shape of the L5 transverse process could be more indicative of pathology than the measurement alone	MRI
Lumbosacral transitional vertebra contributed to lumbar spine degeneration: An MR study of clinical patients	Cheng 2022 [17]	Three hundred and fifty patients with LSTV (52.3 ± 10.9 years), including 182 Castellvi type I, 107 type II, 43 type III, and 18 type IV, and 179 controls without LSTV (50.6 ± 13.1 years), were studied. Discs, endplates, and posterior vertebral structures were assessed and compared to those of controls for the most caudal three discs on MRIs	Cross‐sectional study	To comprehensively characterize degenerative findings associated with various types of lumbosacral transitional vertebra (LSTV) on magnetic resonance images	Castellvi types III and IV LSTV predisposed the adjacent spinal components to degeneration and protected the transitional discs from age‐related degeneration. Type II LSTV had significant effects in promoting transitional and adjacent disc degeneration. Type I LSTV was not related to lumbar spine degeneration	CT
Optimum vertebral level of Castellvi type III or higher lumbosacral transitional vertebrae when measuring spinopelvic parameters	Tatara 2022 [18]	PI and PT were measured twice in 56 patients with type III and IV LSTVs with a balanced spine, with LSTV considered as the lowest lumbar vertebra (LLV) or S1. PI and PT measured with LSTV as LLV were denoted as LLV_PI and LLV_PT, and those measured as S1 were denoted as S_PI and S_PT. The LSTV was interpreted as lowest lumbar vertebra. If LLV_PI, LLV_PT, or both were above the reference range, it was interpreted as S1. If all parameters were within the reference range, it was interpreted as an intermediate type	Cross‐sectional study	To determine the optimum vertebral level of these LSTVs when measuring PI and PT	If the measured PI is less than the reference range, it is likely that the PI was measured with LSTV as S1; and that for this reason, it would be better to remeasure the PI with LSTV as the lowest lumbar vertebra; and conversely, if the measured PT is greater than the reference range despite a high PI and no sagittal imbalance, it is likely that the PT was measured with LSTV as the lowest lumbar vertebra	Mixed
Prevalence of lumbosacral transitional vertebra in individuals with low back pain: Evaluation using plain radiography and magnetic resonance imaging	Shaikh 2017 [19]	All patients aged 11–90 years of either gender with LBP for any duration, who presented for X‐ray and magnetic resonance imaging (MRI) of the lumbosacral spine, were included. X‐rays of the lumbosacral spine in anteroposterior and lateral views were acquired. In addition, T1‐ and T2‐weighted sagittal and axial MRI was performed. Images were evaluated on a workstation	Cross‐sectional study	To determine the frequency of LSTV in patients with low back pain (LBP) and the role of iliolumbar ligament (ILL) origin from L5 in LSTV cases	LSTV occurs at a high frequency in patients with LBP. Furthermore, in the presence of LSTV, the ILL is not a reliable marker for the identification of L5	Mixed
Prevalence of lumbosacral transitional vertebral anomalies among healthy volunteers and patients with hip pathology: association with spinopelvic characteristics	Verhaegen 2023 [20]	This cross‐sectional study included 102 patients with hip pathology and 51 asymptomatic volunteers. Participants underwent radiographic assessment of the lumbar spine and pelvis in standing and deep‐seated positions. LSTV occurrence was classified according to the Castellvi system. Spinopelvic characteristics included lumbar lordosis (including segmental lumbar angles), pelvic tilt, and hip flexion (pelvic‐femoral angle). Differences between standing and deep‐seated values were calculated. Low back pain was assessed using the Oswestry Disability Index	Cross‐sectional study	To determine the (1) LSTV prevalence in young patients presenting with hip pain in comparison with a group of asymptomatic volunteers, (2) effect of LSTVs on static and dynamic spinopelvic characteristics, and (3) presence of low back pain (LBP) among young adult patients with hip pain with an LSTV	An LSTV was found in 8.5% of young adults, with no difference between patients with hip pathology and controls. Individuals with an LSTV have greater standing lumbar lordosis, with altered mechanics at the cephalad adjacent level, which may predispose these individuals to degenerative changes at this level	Radiographs
The association of lumbosacral transitional vertebrae with low back pain and lumbar degenerative findings in MRI: A large cohort study	Hanhivaara 2022 [21]	1468 lumbar spine MRI scans from the NFBC1966 base acquired were assessed for the presence of LSTV and degenerative changes. Castellvi classification was utilized to identify LSTV anatomy. Additionally, 100 controls without LSTV were collected. Self‐reported LBP with a duration of more than 30 days in the past year was deemed clinically relevant. For the statistical analyses, chi square test, independent samples *t* test and multinomial logistic regression analyses were used	Cross‐sectional study	To evaluate the association of lumbosacral transitional vertebrae (LSTV) with low back pain (LBP) and associated degenerative findings using magnetic resonance (MR) imaging	LSTVs were a common finding within this study, and Castellvi type III LSTVs were associated with LBP. Degenerative findings were associated with LSTV anatomy and occurred more commonly above the transitional level. Level of Evidence: 3	MRI
The association of lumbosacral transitional vertebral anomalies with acetabular dysplasia in adult patients with hip‐spine syndrome: A cross‐sectional evaluation of a prospective hip registry cohort	Sun 2021 [22]	Retrospective analysis of registry data, 122 hips in 122 patients who presented with hip pain and a final diagnosis of Acetabular Dysplasia (AD) were studied. Two observers analyzed hip and spine variables using standard radiographs to assess AD. The frequency of lumbosacral transitional vertebra (LSTV), along with associated Castellvi grade, pars interarticularis defect, and spinal morphological measurements were recorded and correlated with radiological severity of AD	Cross‐sectional study	To test the hypothesis that there is a higher frequency of radiological spinal abnormalities in patients with AD and evaluate the relationship between the radiological severity of AD and the frequency of spinal abnormalities	Patients with AD have increased frequency of spinal anomalies seen on standard hip radiographs. However, there exists no correlation between radiological severity of AD and frequency of spine anomalies. In managing AD patients, clinicians should also assess spinal anomalies that are easily found on standard hip radiographs	Radiographs
Lumbosacral transitional vertebral articulation: Evaluation by planar and SPECT bone scintigraphy	Pekindil 2004 [23]	Scintigraphic images were evaluated and blind to the clinical and radiological data. The presence of increased activity in the lumbosacral transitional zone was assessed as focal or non‐focal, and the intensity of activity was classified as normal, minimal, mild, moderate or marked on planar and SPECT images. To assess the degree of uptake intensity, reference region was considered to be the opposite, normal side. The results of the bone scintigraphy were compared with the clinical and X‐ray findings	Cross‐sectional study	To show planar and SPECT bone scintigraphic findings of LSTV and compare them with the LBP and radiographs findings	Focal, markedly increased, uptake may show the metabolically active degenerative changes of LSTV articulation and may help to reveal the pain arising from LSTVA. Therefore the authors propose that bone scintigraphy may be considered for the evaluation of patients with LBP thought to arise from LSTV articulation	Scintigraphy
Castellvi classification of lumbosacral transitional vertebrae: Comparison between conventional radiography, CT, and MRI	Hanhivaara 2024 [24]	Retrospective cross‐sectional study, a total of 852 patients undergoing lumbar imaging studies using all three modalities were initially assessed for the presence of LSTV using CT scans. In total, 100 patients with LSTV anatomy were identified. Four readers performed blinded and independent evaluations of these 100 patients on each modality, and an experienced fellowship‐trained radiologist performed a gold standard read using all three modalities	Cross‐sectional study	To compare the diagnostic performance of conventional radiography (CR), computed tomography (CT), and magnetic resonance imaging (MRI) in classifying LSTVs	CT had the highest diagnostic performance in all measured metrics with good inter‐reader reliability. MRI and CR showed fairly poor sensitivity and accuracy, and thus consideration should be used when classifying LSTVs with these two modalities	Mixed
A computed tomography vertebral segmentation dataset with anatomical variations and multi‐vendor scanner data	Liebl 2021 [26]	Retrospective evaluation of imaging data. Inclusion criteria for the dataset: Subjects older than 18 years were included, who had received CT imaging of the spine showing a minimum of seven fully visualized vertebrae without counting sacral vertebrae or transitional vertebrae. The final dataset comprised 300 subjects. All selected imaging series were categorized regarding their primary attribute	Retrospective study	To enrich the “Large Scale Vertebrae Segmentation Challenge” VerSe 2020 dataset with rare anatomical variants to improve derived model performance	The successful segmentation challenges held at the MICCAI conferences in 2019 and 2020 based on these public datasets confirm that algorithms for segmentation of the spine can be trained and that algorithm performance benefits from large and diverse datasets	CT
Analysis of spinopelvic parameters and lumbar lordosis in patients with transitional lumbosacral vertebrae, with special reference to sacralization and lumbarization	Karabag 2024 [27]	Abdominal CT scans were performed; all scans were obtained with the patient in a standard supine position with knees extended. Of the 1420 patients included, 108 (cumulative group) patients had Castellvi Type 2a or above. The spinopelvic parameters were measured. The thoracic vertebrae corresponding to the ribs were counted to locate the L1 vertebra. The first vertebra without a costal articulation was accepted as the L1 vertebra; for patients with a short or hypoplastic rib, or a hypoplastic or absent transverse process, the L1 vertebra was determined according to the proximal psoas insertion	Retrospective study	To evaluate spinopelvic parameters in asymptomatic patients with sacralization and lumbarization and compare them with each other and normative values	Upper and lower endplate parameters are comparable in patients with sacralization and lumbarization; therefore, the average spatial position of a sacralized L5 and a lumbarized S1 within the pelvis is similar and either parameter can be used for radiological measurements	CT
A simple method for the selection of valid spinopelvic parameters and lumbar lordosis in patients with transitional lumbosacral vertebrae	Iplikcioglu 2024 [28]	Upper and lower endplate spinopelvic parameters (i.e., pelvic incidence [PI], sacral slope [SS], and pelvic tilt) and LL of 108 patients with TLSV were measured by computed tomography. In addition, these parameters were measured for randomly selected subjects without TLSV. The PI value in the TLSV group, which was closer to the mean PI value of the control group, was accepted as valid and then used to create an optimum PI (OPI) group. Finally, the spinopelvic parameters and LL of the OPI and control groups were compared	Retrospective study	To describe a standardization method for measuring the spinopelvic parameters and LL in patients with TLSV	In patients with TLSV, the endplate associated with a PI value closer to the mean normative PI value can be selected as the reference line to measure the spinopelvic parameters and LL. This finding indicates that normative values for spinopelvic parameters and LL can serve as a criteria for diagnosis and surgical indications in patients with TLSV	CT
Changes in lumbosacral anatomy and vertebral numbering in patients with thoracolumbar and/or lumbosacral transitional vertebrae	Tatara 2021 [29]	Retrospective revision of 880 patients who underwent spinopelvic fixation between July 2014 and March 2020 were evaluated for TLTV and LSTV. All patients underwent whole‐spine radiography, CT, and MRI preoperatively. LSTV was observed in 111 (12.6%) of 880 patients. The number of presacral vertebrae was counted caudally from C2 using whole‐spine radiographs. LSTV was detected on three‐dimensional CT images and classified according to the Castellvi. The anatomical location of the lowest thoracic vertebra was defined using coronal CT images, and TLTV with dysplastic ribs was identified. Each LSTV type was examined for its morphological features on sagittal and axial CT images	Retrospective study	To examine the rib morphology of the lowest thoracic vertebra to identify TLTV using CT images and to investigate the incidence of TLTV and LSTV	Although LSTV possesses L5 and S1 features, Castellvi LSTVs have more L5 elements than S1 elements. The converse is true for S6 LSTV. At least for the Castellvi type‐IIIb LSTV, the vertebra below the Castellvi type‐IIIb LSTV should be recognized as S1, but clinically it is better to recognize it as S2. The three‐dimensional CT images are suitable for detecting transitional vertebrae	Mixed
Determining the best vertebra for measuring pelvic incidence and spinopelvic parameters in adult spinal deformity patients with transitional anatomy	Ani 2023 [30]	Multicenter retrospective comparative cohort study comprising a large database of adult spinal deformity (ASD) patients and a database of asymptomatic individuals. Linear regression modeling was used to determine normative T1 pelvic angle (TPA) and PI—lumbar lordosis (LL) mismatch (PI‐LL) based on PI and age in a database of asymptomatic subjects. In an ASD database, patients with radiographic evidence of L5 sacralization had the PI, LL, and TPA measured from the superior endplate of S1 and then also from L5	Retrospective study	To determine if spinal deformity patients with L5 sacralization should have pelvic incidence (PI) and other spinopelvic parameters measured from the L5 or S1 endplate	Measuring the PI and spinopelvic parameters at L5 in sacralized anatomy results in underestimating spinal deformity and is less correlated with health‐related quality of life. Surgeons may consider measuring PI and spinopelvic parameters relative to S1 rather than at L5 in patients with a sacralized L5	Radiographs
Does lumbosacral transitional vertebra have any influence on sacral tilt?	Benlidayi 2015 [31]	1588 anterioposterior and lateral lumbar radiographs of patients with low back pain performed between March 2013 and September 2013 were extracted from the medical electronic database. Among these radiographs, those belonging to patients with Castellvi types II, III, and IV LSTV were identified. The angle of ST was measured on lateral lumbar radiographs and compared with that of age‐ and sex‐matched controls without LSTV	Retrospective study	To compare the sacral tilt (ST) angle between patients with and without lumbosacral transitional vertebra (LSTV)	In conclusion, the study showed that ST, which delineates the sagittal spatial orientation of sacrum is smaller in patients with LSTV than those without any transitional vertebral anomaly. Because the radiographic data was derived from the electronic medical database, image quality was not in a standard manner for all radiographs	Radiographs
Effect of lumbosacral transitional vertebrae on sagittal balance of lumbo‐pelvic complexity assessed by quantitative whole‐body CT imaging	Zhou 2023 [32]	Retrospective reviewed CT images of 6097 Chinese patients who underwent whole‐body positron emission tomography combined with computed tomography (PET/CT) scans from October 2017 to December 2019. Musculoskeletal radiologist reviewed all whole‐spine CT images with 3D volume rendered and multiplanar reconstruction techniques to identify LSTV. A total of 210 individuals with complete segmentation anomalies of LSTV were included and divided into 23 presacral vertebrae with sacralization (*n* = 102) and 25 with lumbarization (*n* = 108). Sagittal lumbo‐pelvic parameters were measured at the ontogenetical S1 (Ontog S1) level and the morphological S1 (Morph S1), respectively	Retrospective study	To investigate the effect of LSTV on the assessment of sagittal lumbo‐pelvic balance and provide some recommendations for the preoperative imaging evaluation	Morph S1 is more recommended for the measurements of most lumbo‐pelvic parameters in patients with LSTV. The parameters (PT, SS, LL, STA, PR) are shown more stable and recommended to help reduce the effects caused by LSTV	PET‐CT
Evaluation of spinal‐paraspinal parameters to determine segmentation of the vertebrae	Peker 2019 [33]	143 Patients who underwent MRI or computed tomography scans of the whole cervical, thoracic, and lumbar spine were included in the study. Vertebra corpus shape, O'Driscoll classification, lumbosacral axis angle, last two square vertebra dimensions, orifice of right renal artery (RRA), orifice of celiac truncus (CT), orifice of superior mesenteric artery (SMA), vena cava inferior confluence (CVC), abdominal aorta bifurcation (AB), and iliolumbar ligament were evaluated in this study	Retrospective study	To evaluate whether lumbar vertebrae can be correctly numbered using auxiliary parameters	According to the results of the study, no single parameter in the magnetic resonance imaging can accurately indicate the number of vertebrae without counting the levels. As a result, the authors believe that these parameters may be suspicious in terms of the presence of LSTV rather than the correct level	MRI
Evaluation of the role of anatomical landmarks in lumbosacral transitional anomalies identification on magnetic resonance imaging of spine	Garg 2021 [34]	260 patients with confirmed L5 level (identified on 1.5 T MR screening of whole spine) were included in the study. The level(s) of ILL, CF, AB, PM and CL were documented in all of them, and analysis was done	Retrospective study	To assess usefulness of different anatomical structures (ILL, CF, AB, PM and CL), that can help in counting and identifying last lumbar vertebrae in cases of a LSTV	ILL, CF, AB, PM and CL had variable origin with caudal and cranial shifts in lumbarization and sacral‐ization respectively and these are not reliable identifier of the L5 vertebra in the setting of LSTV anomalies	MRI
Examining degenerative disease adjacent to lumbosacral transitional vertebrae: A retrospective cohort study	Desai 2023 [35]	Retrospective comparison of patients with Bertolotti syndrome and control patients with chronic back pain with no LSTV. The presence of an LSTV was confirmed on imaging, and the caudal‐most mobile segment above the LSTV was assessed for degenerative changes. Degenerative changes were assessed by grading the intervertebral disc, facets, degree of spinal stenosis, and spondylolisthesis using well documented grading systems	Retrospective study	This study examined degenerative changes at segments superjacent to the LSTV in patients with Bertolotti syndrome	Bertolotti patients had a significantly higher PI and were more likely to have adjacent‐segment disease (ASD; L4–5). However, after controlling for age and sex, PI and ASD did not appear to have a significant association within the cohort of Bertolotti patients. This association may warrant closer follow‐up protocols for patients being treated for Bertolotti syndrome, but further prospective studies are needed	CT
Influence of lumbosacral transitional vertebrae on spinopelvic parameters using biplanar slot scanning full body stereoradiography‐analysis of 291 healthy volunteers	Okamoto 2022 [36]	A total of 291 healthy adult volunteers with no history of spinal disease were evaluated with stereoradiography to determine the prevalence of LSTV. Vertebrae were counted from the first cervical vertebra using both coronal and sagittal plane images. It was investigated the influence of LSTV on whole‐body sagittal alignment in 279 participants. Whole‐body key parameters descriptive statistics were compared among groups according to the number of vertebrae (L4, L5, and L6). Statistical analysis was performed between normal and LSTV cases using the Steel‐Dwass analysis	Retrospective study	To prove that the location of the sacral base differs between populations with LSTV and normal populations and to investigate the effects of lumbosacral transitional vertebrae anomalies on whole‐body sagittal alignment	The authors propose a precise method for numbering the vertebrae using coronal and sagittal full body images. The spinopelvic parameters of the LSTV population significantly differed from those in the normal spine population due to differences in the sacral base location	EOS
Interreader and intermodality reliability of standard anteroposterior radiograph and magnetic resonance imaging in detection and classification of lumbosacral transitional vertebra	Farshad‐Amacker 2014 [37]	Retrospective case control study. Review board approval, coronal MRI scans and conventional AP radiographs of 155 subjects (93 LSTV type 2 or higher and 62 controls) were retrospectively reviewed by two independent, blinded readers and classified according to the Castellvi classification. Interreader reliability was assessed using kappa statistics for detection of an LSTV and identification of all subtypes for MRI scans and standard AP radiographs. Further, accuracy and positive and negative predictive values were calculated for standard AP radiographs to detect and classify LSTV using MRI as the gold standard	Retrospective study	To evaluate the interreader reliability of detection and classification of LSTV with standard AP radiographs and report its accuracy by use of intermodality statistics compared with MRI as the gold standard	Standard AP radiographs are insufficient to detect or classify LSTV. Coronal MRI scans, however, are highly reliable for classification of LSTV	Mixed
Localizing the L5 vertebra using nerve morphology on MRI: An accurate and reliable technique	Peckham 2017 [38]	108 cases with full spine MRI were numbered from the C2 vertebral body to the sacrum with note of thoracolumbar and lumbosacral transitional states. The origin level of the L5 nerve and iliolumbar ligament were documented in all cases. The reference standard of numbering by full spine imaging was compared with the nerve morphology numbering method. Prevalence and bias‐adjusted *κ* were used to measure interrater and intrarater reliability	Retrospective study	To determine whether MRI morphologic features of the lumbar nerves could be used to distinguish the lower lumbar levels and to apply these characteristics in localizing the L5 vertebra	The exiting L5 nerve can allow accurate localization of the corresponding vertebrae, which is essential for preprocedural planning in cases where full spine imaging is not available	Mixed
Lumbar plain radiograph is not reliable to identify lumbosacral transitional vertebra types according to Castellvi classification principle	Hou 2020 [39]	Patients with suspected LSTV determined by AP lumbar radiographs were initially enrolled. Among them, those who received CT were formally enrolled to verify the sensitivity of AP lumbar radiography on detecting and classifying LSTV types according to the Castellvi classification principle	Retrospective study	To verify the sensitivity and specificity of AP‐LPR to detect and classify MA‐LSTV types according to the Castellvi classification principle, using CT‐CRIs at the bone window from a 256‐slice helical CT machine as the gold standard	Although AP‐LPR could correctly detect MA‐LSTV, it could not give accurate type classification. CT‐CRIs could provide detailed information between the TP and sacrum area and could be taken as the gold standard to detect and classify MA‐LSTV	Mixed
Lumbosacral transitional vertebrae alter the distribution of lumbar mobility‐preliminary results of a radiographic evaluation	Becker 2022 [40]	A retrospective study of 51 patients with osteochondrosis L5/S1 with flexion and extension radiographs was performed. 17 patients had LSTV. The lumbar and segmental range of motion by segmental lordosis angle and the segmental wedge angle were determined. Parametric data were compared by paired *T*‐test. Non‐parametric data were compared by Wilcoxon‐rank‐sum‐test. Correlations were observed using Spearman's Rank correlation coefficient. A *p*‐value < 0.05 was stated as statistically significant	Retrospective study	To investigate the mobility of the lumbar spine and segmental motion distribution of patients with LSTV in flexion‐extension radiographs	Patients with LSTV show a reduced RoM in the transitional segment and a significantly increased motion distribution to the cranial adjacent segment in flexion‐extension radiographs. The increased proportion of mobility in the cranial adjacent segment possibly explain the higher rates of degeneration within the segment	EOS
Lumbosacral transitional vertebrae: An overlooked cause of back pain on MRI	TÜRK 2023 [41]	All anteroposterior radiographs were evaluated regarding the presence of LSTV, while ST angle was measured on lateral lumbosacral radiographs. Patients with types II, III and IV LSTV based on Castellvi method were included into the study, while those with LSTV I were excluded	Retrospective study	To evaluate the presence of LSTV in patients who underwent MRI with a pre‐diagnosis of sacroiliitis	No correlation was found between gender or presence of sacroiliitis and any type of LSTV LSTV may present with backpain and should be considered in patients where sacroiliitis is clinically suspected. MRI is a useful tool to identify other accompanying pathologies in these cases	MRI
Lumbosacral transitional vertebrae are associated with lumbar degeneration: Retrospective evaluation of 3855 consecutive abdominal CT scans	Hanhivaara 2020 [42]	A total of 3855 abdominal CT scans of the year 2017 from a single hospital were retrospectively assessed for LSTV, disc degeneration (DD), and facet joint degeneration (FD). An age‐ and sex‐matched 150‐subject control group without LSTV was picked at random. Multivariable logistic regression was used for the analysis	Retrospective study	To assess the prevalence of LSTV and associated spinal degenerative changes on abdominal CT scans in Caucasian population	LSTV Castellvi types II, III, and IV are associated with greater lumbar degeneration, warranting meticulous evaluation of spinal anatomy, even on CT	CT
Musculature adaption in patients with lumbosacral transitional vertebrae: A matched‐pair analysis of 46 patients	Becker 2021 [43]	Abdomen–pelvis CT scans were analyzed in patients with LSTV and a matched control group. LSTV were classified according to the Castellvi classification. Muscles were segmented from the remaining soft tissue and their cross‐sectional area and volume were examined at five defined levels. For comparison of categorical data, chi‐squared tests were performed and for associations between the degree of fusion and muscle size and degeneration, Spearman's correlation coefficients were calculated. Inter‐ and intrarater reliabilities were evaluated by computing intraclass correlation coefficients	Retrospective study	To analyze the association between LSTV and changes in volume, mass, symmetry, and degeneration of lumbar and trunk muscles	LSTV are associated with a reduction in muscle volume and an increase in muscle degeneration of both lumbar and trunk muscles	CT
Prevalence of lumbosacral transitional vertebra among 4816 consecutive patients with low back pain: A computed tomography, magnetic resonance imaging, and plain radiographic study with novel classification schema	Byvaltsev 2023 [44]	During the period from 2007 to 2017, all cases of LSTV were preoperatively verified, and classified according to Castellvi, as well as O'Driscoll. Authors developed modifications of those classifications that are simpler, easier to remember, and clinically relevant. At the surgical level, this was assessed intervertebral disc and facet joint degeneration	Retrospective study	To assessment of the prevalence of lumbosacral transitional vertebra (LSTV) in patients with low back pain and the development of clinically relevant classification to describe these anomalies	LSTV is a common pathology of the lumbosacral junction, occurring in 8.1% of the patients in our series (389 out of 4816 cases). The most common types were Castellvi's type IIA (30.9%) and IIIA (34.9%) and were O'Driscoll's III (40.1%) and IV (35.8%)	Mixed
Quantitative measurements at the lumbosacral junction are more reliable parameters for identifying and numbering lumbosacral transitional vertebrae	Zhou 2022 [45]	A total of 2845 PET/CT scans were reviewed, and the patients with 23 and 25 presacral vertebrae were included. The quantitative parameters, including the anterior‐edge vertebral angle of the lowest lumbar‐type vertebra, the ratio of the length of the inferior endplate to that of the superior endplate of the uppermost sacral‐type vertebra and the lumbosacral intervertebral disc angle, and the anatomical landmarks, including the iliac crest tangent level, the iliolumbar ligament origin level and psoas proximal insertion, were all evaluated to determine their ability to identify LSTV	Retrospective study	To evaluate quantitative parameters to identify the anatomic variation lumbosacral transitional vertebrae of and compare them with the landmarks commonly used at present	Compared with the anatomical landmarks, the quantitative measurements at the lumbosacral junction, including anterior‐edge vertebral angle of the lowest lumbar‐type vertebra and ratio of the length of the inferior endplate to that of the superior endplate, may be more helpful for differentiating subgroups of LSTV especially if only lumbar spine imaging is available	PET‐CT
Role of iliac crest tangent in correct numbering of lumbosacral transitional vertebrae	Gündüz 2019 [46]	58 patients with LSTV and 55 controls without LSTV who had undergone spinal computed tomography were included. The iliac crest tangent (ICT) was drawn on the coronal images, with the cursor in the sagittal view set to the posterior ⅓ of the vertebral body located one level above the LSTV. When more than 1.25 vertebral body was counted below the ICT, the LSTV was considered as S1, otherwise it was considered as L5. The gold standard was counting the vertebrae craniocaudally	Retrospective study	To evaluate the value of the ICT as a landmark in patients without disc degeneration	Iliac crest tangent (ICT) does not seem to be a reliable landmark for correct numbering of LSTV in patients with no intervertebral disc degeneration	CT
Sacroiliac joint variations on magnetic resonance imaging in patients with low back pain	Özbalcı 2022 [47]	Retrospective study included all Sacroiliac Joint (SIJ) MRI examinations performed in our hospital with patients ≥ 18 and < 65 years of age for 24 months. Data collection consisted of the patients age at the imaging time, gender, and the presence of active and chronic sacroiliitis. LSTV was classified according to the Castellvi classification system. Moreover, all images were assessed for the presence of major sacroiliac joint variations described in the literature. Structural and edematous changes were also noted	Retrospective Study	To investigate the frequency of normal anatomical variations and to reveal their importance by distinguishing the findings that mimic sacroiliitis in patients referred to MRI for LBP	The lumbosacral transition segments and various anatomical SIJ variations are common in the low back pain population, especially in women. Moreover, these variations may be associated with degenerative and edematous signal intensity changes that mimic sacroiliitis	MRI
The prevalence and clinical significance of transitional vertebrae: A radiologic investigation using whole spine spiral three‐dimensional computed tomographic images	Doo 2020 [48, 49]	The vertebral levels were counted craniocaudally, starting from C1, based on the assumption of 7 cervical, 12 thoracic, and 5 lumbar vertebrae, using whole spine spiral three‐dimensional computed tomographic images. The 20th and 25th vertebrae were defined as L1 and S1, respectively	Retrospective study	To investigate the prevalence of TLTV and LSTV (lumbarization and sacralization) by evaluating whole spine CT. And the authors evaluated the relationships between the existence of TLTV and the existence of abnormal rib count as the primary outcome and the correlation between the existence of thoracolumbar and lumbosacral transitional vertebra as the secondary outcome	The study results suggest that patients with TLTV are more likely to have an abnormal rib count or LSTV. If a TLTV or LSTV is seen on the fluoroscopic image, a whole spine image is necessary to permit accurate numbering of the lumbar vertebra	CT
Variations in the number of vertebrae, prevalence of lumbosacral transitional vertebra and prevalence of cervical rib among surgical patients with adolescent idiopathic scoliosis: An analysis of 998 radiographs	Chiu 2024 [49]	This was a retrospective study on adolescent idiopathic scoliosis AIS patients who underwent posterior spinal fusion. Demographic and anthropometric data, radiographic data (Lenke curve type, pre‐operative Cobb angle, vertebra numbering of cervical, thoracic, and lumbar spine, presence of LSTV based on the Castellvi classification and the presence of cervical ribs) and clinical data were collected. Multinomial logistic regression analyses were performed to identify factors associated with the outcomes of interest	Retrospective study	To investigate variation in the number of thoracic and lumbar vertebrae, the prevalence of lumbosacral transitional vertebra (LSTV) and the prevalence of cervical ribs among surgical patients with adolescent idiopathic scoliosis (AIS)	Seven different variations in the number of cervical, thoracic, and lumbar vertebrae were identified. The total prevalence of patients with atypical vertebrae variation was 15.5%. LSTV was found in 25.1% of the cohort. Due to the differences in the number of morphologically thoracic and lumbar vertebrae, there may still be a risk of inaccurate identification	Radiographs
Interpretation of spinal radiographic parameters in patients with transitional lumbosacral vertebrae	Zhou 2018 [50]	Cases series. Patients with LSTV were identified and radiographic spinopelvic measurements were obtained. Radiographic measurements were performed twice with the sacral endplate at the cephalad and caudal options. Paired *t* tests assessed the difference between different selection groups	Retrospective study	To understand the effect of variability in sacral endplate selection in transitional lumbosacral vertebrae (TLSV) and its impact on pelvic, regional, and global spinal alignment parameters	Variation in sacral endplate selection in LSTV significantly affects spinal alignment parameter measurements. A standardized method for measuring LSTV is needed to reduce measurement error and ultimately allow more accurate understanding of alignment targets in patients with LSTV	EOS
Effect of spinal segment variants on numbering vertebral levels at lumbar MR imaging	Carrino 2011 [51]	A review of 147 subjects was performed by using spine radiography as the reference standard to determine total and segmental vertebral count and transitional anatomy. S1–2 disk morphology was rated according to the classification by O'Driscoll et al., and the ILL level was determined from MRI	Retrospective study	To verify ILL location, to evaluate MRI morphologic features for detecting LSTV, and to determine whether transitional situations are associated with anomalous vertebral numbering	The ILL denotes the lowest lumbar vertebra, which does not always represent L5. A well‐formed, complete S1–2 intervertebral disk is associated with LSTV. LSTV are associated with anomalous vertebral numbering	Mixed
Prediction of transitional lumbosacral anatomy on magnetic resonance imaging of the lumbar spine	Chalian 2012 [52]	Retrospective Study. The lumbar spine MRI studies of 50 subjects with LSTV and 50 subjects with normal lumbosacral anatomy were retrospectively evaluated. In each study, the mid‐sagittal T2‐weighted image was used to measure the angle formed by a line parallel to the superior surface of the sacrum and a line perpendicular to the axis of the scan table, as well as the angle formed by a line parallel to the superior endplate of the L3 vertebra and a line parallel to the superior surface of the sacrum	Retrospective study	To evaluate two simple angle measurements for predicting lumbosacral transitional vertebra (LSTV) in magnetic resonance imaging (MRI) studies of the spine	On sagittal MR images of the lumbar spine, an increased A‐angle and/or B‐angle should alert the radiologist to the presence of LSTV	MRI
Numbering of lumbosacral transitional vertebrae on MRI: Role of the iliolumbar ligaments	Hughes 2006 [53]	Five hundred consecutive lumbar spine MRI studies were reviewed. A standard protocol of sagittal and axial T1‐weighted and T2‐weighted spin‐echo sequences was used. The sagittal images were assessed for the presence of an LSTV, and axial images were assessed for the level of origin of the iliolumbar ligaments	Retrospective study	To determine whether identification of the iliolumbar ligaments is of practical use for numbering lumbosacral transitional vertebrae (LSTV)	The iliolumbar ligament is readily identifiable on axial lumbar spine MRI and always arises from L5. We suggest that its position can be used to confidently assign lumbar levels in patients with LSTV	MRI
Effect of lumbosacral transitional vertebra on developmental alterations of the hip: A quantitative investigation of the lumbo‐pelvic‐hip complex via whole‐body computed tomography	Luo 2024 [54]	A total of 310 individuals were categorized into three groups according to whole‐body computed tomography (CT) imaging: a group with sacralization of 23 presacral vertebrae (PSV) (*n* = 102), a group with lumbarization of 25 PSV (*n* = 108), and a normal control group with 24 PSV (*n* = 100). Quantitative parameters of the lumbo‐pelvic‐hip complex were measured and analyzed	Retrospective study	To investigate the impact of LSTV on developmental alterations of the hip	Variations of LSTV are correlated with the hip anatomical development via lumbo‐pelvic‐hip complex transmission and may potentially reduce the sagittal acetabular coverage, particularly in the 23 PSV subtype on the right side	CT

### 3.1 Computed Tomography (CT)

Ten studies published between 2019 and 2024 evaluated the diagnosis of LSTV using CT. Of these, nine [26, 27, 28, 35, 42, 43, 46, 48, 54] studies were retrospective, and one was a cross‐sectional study [17]. These articles describe the use of CT, whether thoracic/abdominal or pelvic. Among the radiological findings to consider are the use of anatomical landmarks such as the superior vena cava, spinopelvic parameters, rib, and thoracic vertebra counts.

Iplikcioglu and Karabg [27, 28] conducted two studies with the same cohort. The authors performed a retrospective study using abdominal CT scans of 1420 patients, of whom 108 had LSTV. The diagnosis was established by identifying unilateral or bilateral pseudoarticulation or complete fusion between the transverse process of the lowest lumbar vertebra and the sacrum, following the Castellvi classification. Both studies emphasized the measurement of pelvic parameters such as pelvic incidence, pelvic tilt, sacral slope, lumbar lordosis, and the difference between pelvic incidence and lumbar lordosis using the superior and inferior endplates of the transitional vertebra. Significant differences were found between upper and lower endplate measurements, although both were highly correlated, and the authors proposed the concept of an “optimum pelvic incidence,” defined as the endplate that yields a value closest to the mean of normal subjects. These findings suggest that LSTV morphology can influence pelvic alignment and that careful selection of the endplate is relevant for accurate measurement.

Similarly, Liebl et al. [26] conducted a retrospective study, including 300 patients with CT images of the spine from four scanner providers. In this study, they aimed to address the large‐scale vertebral segmentation challenge. For the diagnosis of LSTV, they used the well‐established Castellvi classification for LSTV, primarily based on the morphology of the transverse process of the last lumbar vertebra and whether it was fused with the sacrum. Before staging the degree of LSTV, they analyzed the count and variants of cervical (n: 1 and 2), thoracolumbar (n: 11, 12, and 13), and lumbosacral (n: 4, 5, and 6) vertebrae. The study highlighted the importance of complete spinal visualization to ensure accurate classification and labeling. Anatomical variants were frequent, with 33.7% of cases showing six lumbar vertebrae and 28% showing short ribs, reinforcing the high frequency of segmentation anomalies associated with transitional vertebrae. In the study conducted by Doo et al. [48], also retrospective, they evaluated 1553 whole‐spine CTs to determine the prevalence and relationship between thoracolumbar and lumbosacral transitional vertebrae. Regarding imaging, the following parameters were used: first, vertebral levels were counted cranio‐caudally, using 3D‐CT images of the entire spine from C1, assuming 7 cervical, 12 thoracic, and 5 lumbar vertebrae. Vertebrae 20 and 25 were defined as L1 and S1, respectively. Several investigators proposed that most caudal ribs could be classified into one of four types: normal rib, hypoplastic rib (or short rib), non‐fused transverse process (or accessory ossification center), and mixed type. Sacralization was defined as the abnormal fusion of L5 (the 24th vertebra) with S1. They found a prevalence of 11.2% for thoracolumbar and 8.3% for LSTV, both frequently associated with abnormal rib counts. Patients with thoracolumbar transitional vertebrae were seven times more likely to have LSTV compared with those without this anomaly. The authors concluded that complete spinal imaging is essential for accurate vertebral numbering, as partial counts based on ribs or fluoroscopic references can easily lead to errors.

Expanding on functional implications, Luo et al. [54] conducted another retrospective study aimed at investigating the impact of LSTV on hip development alterations. In this study, 310 individuals were categorized into three groups based on full‐body CT: a group with sacralization of 23 vertebrae, a group with lumbarization of 25 presacral vertebrae, and a normal control group with 24 presacral vertebrae. All were diagnosed with LSTV via CT. They found that LSTV significantly modified pelvic parameters such as pelvic incidence, pelvic tilt, sacral slope, and lumbar curvature, as well as acetabular morphology. Sacralization was associated with increased sagittal acetabular anteversion and reduced sagittal acetabular coverage, suggesting that LSTV alters lumbopelvic and hip biomechanics. These findings highlight that transitional anatomy should be considered during hip replacement planning to ensure correct implant positioning.

Degenerative changes associated with LSTV were a focus of multiple studies. Hanhivaara et al. [42] conducted a retrospective study using 3855 abdominal CTs and reported a prevalence of 28.6%. Castellvi types II–IV were associated with higher rates of degeneration in intervertebral discs and facet joints, particularly at the L4–L5 level. The authors also proposed two new Castellvi subtypes (IIc and IIIc) to describe unilateral pseudoarticulation or fusion with contralateral transverse process enlargement and reported excellent interobserver agreement. Supporting these findings, the cross‐sectional study conducted by Cheng et al. [17] examined 529 patients, 350 of whom had LSTV confirmed by CT and MRI. LSTV was classified as type I–IV and then marked as “a” if unilateral or “b” if bilateral and classified according to Castellvi. The authors observed that Castellvi type I was not related to degenerative changes, while type II was associated with degeneration affecting both the transitional and adjacent discs. In contrast, types III and IV showed less degeneration at the fused transitional level but more advanced changes in the cranial segment, including disc protrusion, endplate defects, and spondylolisthesis. These results support that higher Castellvi types predispose to adjacent‐segment degeneration, like what occurs after surgical fusion, whereas type II presents a mixed pattern involving both the transitional and adjacent levels. Additionally, in the study performed by Desai et al. [35], a retrospective comparison was made of 172 patients diagnosed with Bertolotti's syndrome (*n* = 101) or lumbar spondylosis without identified LSTV (*n* = 71) documented in the clinical history or present in the images. Diagnosis was confirmed by CT following the Castellvi classification, and the study demonstrated that patients with LSTV had higher pelvic incidence and greater adjacent‐segment degeneration at L4–L5 compared with controls. However, after adjusting for age and sex, these associations were no longer significant, suggesting that both anatomical and biomechanical factors contribute to the degenerative process.

Another dimension was explored in musculature‐related changes. In the article authored by Becker et al. [43], a retrospective study was conducted, and 46 matched controls using abdominal and pelvic CT to evaluate muscle morphology. Patients with LSTV showed lower paraspinal and abdominal muscle volume and increased fatty degeneration, except in the most caudal paravertebral muscles. These findings suggest that LSTV leads to muscular atrophy and changes in lumbopelvic biomechanics. Lastly, Gündüz et al. [46] conducted a retrospective study including 113 patients, 58 of whom had LSTV, with the goal of evaluating the reproducibility and accuracy of the iliac crest tangent as a reference point in subjects without disc degeneration. In this study, they also used full‐spine CT for the diagnosis of LSTV. The number of LSTV was evaluated according to the iliac crest tangent measurement without prior assessment of the correct LSTV count. The results concluded that the iliac crest tangent does not appear to be a reliable reference point for the correct numbering of LSTV in patients without intervertebral disc degeneration.

### 3.2 PET‐CT

Four articles were identified in which the diagnosis of LSTV was made using PET‐CT. These articles were published between 2017 and 2023. Of these, there was one case report [10], one case series [9], and two retrospective studies [32, 45].

The studies reported by Usmani et al. [9, 10] involved a case report and a case series with 55 patients, both symptomatic and asymptomatic, diagnosed with incidental LSTV who underwent PET‐CT with 18F‐NaF. Of the 34 symptomatic patients, most showed focal uptake of the tracer at the pseudoarticulation site, demonstrating a linear trend between the intensity of the uptake and the presence of symptoms. Therefore, the degree of uptake was mentioned as a potential biological marker of symptomatic LSTV. Moreover, 18F‐NaF PET‐CT showed high diagnostic performance, with a sensitivity of 82% and a specificity of 86% in differentiating symptomatic from asymptomatic patients. The authors concluded that 18F‐NaF PET‐CT is a sensitive tool for detecting areas of abnormal bone remodeling and provides molecular insight into the biomechanical alterations caused by LSTV [9].

Zhou et al. [32, 45] conducted two retrospective studies using full‐spine CT from PET/CT for the diagnosis of LSTV. In the first study, which included 6097 patients, 210 were diagnosed with LSTV. The authors found that parameters such as pelvic tilt, sacral slope, lumbar alignment, sacral table angle, and pelvic radius were stable and reliable for assessing sagittal balance in LSTV cases, particularly when measured using the Morph S1 reference. They also identified vertebral count as an independent factor influencing sagittal pelvic parameters. In the second study, they reviewed 2845 patients who underwent PET/CT under the same protocol, with 222 included for analysis. They compared new quantitative metrics, such as the anterior‐edge vertebral angle and the ratio of inferior‐to‐superior endplate length, with traditional anatomical landmarks like the iliac crest tangent, iliolumbar ligament, and psoas insertion. The quantitative parameters achieved higher diagnostic accuracy (around 90%) and reproducibility than conventional markers, making them more useful for distinguishing between L5 sacralization and S1 lumbarization in CT imaging.

### 3.3 Scintigraphy

Two articles were identified in which the diagnosis of LSTV was made using bone scintigraphy. These articles were published between 2004 and 2015. Of these, there was one case report [11] and one cross‐sectional study [23].

Kassir et al. [11] published a case report where they diagnosed LSTV using single‐photon emission computed tomography (SPECT) in a 16‐year‐old woman with lumbar pain lasting for 8 months, for which a bone scan was requested due to suspicion of a pars interarticularis defect. The results showed a focus of increased radiotracer activity in the left lumbosacral area over the left sacroiliac joint, which was consistent with LSTV type IIa. The authors concluded that whole‐body bone scintigraphy with hybrid SPECT/CT imaging of the painful area can be a useful tool in diagnosing LSTV.

Pekindil et al. [23] conducted a retrospective cross‐sectional study diagnosing LSTV through SPECT/CT in patients previously diagnosed by radiography. Changes in their conditions were analyzed to compare both imaging findings. The study included 28 patients, and the results showed that planar scans demonstrated mild to normal or non‐focal uptake with mild to moderate increase, while SPECT demonstrated focal uptake from mild to moderate and significantly increased in patients with degenerative changes without lumbar pain and with lumbar pain, respectively. Radiographs showed an association between degenerative changes and lumbar pain, and SPECT results showed focal uptake that was markedly increased. They concluded that bone scintigraphy can be considered for evaluating patients with lumbar pain believed to arise from the LSTV joint.

### 3.4 Magnetic Resonance Imaging (MRI)

Nine articles were identified that made the diagnosis of LSTV using MRI. These articles were published between 2006 and 2024. Among these, there were six retrospective studies [33, 34, 41, 47, 52, 53] and three cross‐sectional studies [16, 21, 25].

Several studies addressed the diagnostic reliability and anatomical challenges of identifying LSTV using MRI. In a cross‐sectional study performed by Landauer et al. [16], 1842 patients were radiologically evaluated and underwent an MRI of the lumbosacral junction. Of the total patients, 115 were diagnosed with Castellvi II–IV and compared with the literature, noting that Castellvi I was excluded from the study. The authors mentioned that the diagnosis of LSTV with only spinal radiography, without a Ferguson view, is ambiguous, and standard lumbar spine radiographs, as well as spinal radiographs, do not provide a reliable orthogonal representation of the transverse process of L5. Since Ferguson view is not taken for radiological hygiene, they state that the alternative is MRI, which provides additional information in all three planes of space. They concluded that the shape of the L5 transverse process might be more indicative of pathology than just the measurement alone, supporting the association between LSTV and LBP.

Similarly, Peker et al. [33] carried out a retrospective study of 143 patients who underwent MRI or CT of the entire spine to evaluate if lumbar vertebrae can be properly numbered using auxiliary parameters. The authors used only MRI to diagnose LSTV in 13 patients based on vertebral morphology and the lumbosacral angle. Vertebral numbering began from the C2 vertebra, counting seven cervical vertebrae and 12 thoracic vertebrae downwards. If there were no differences between the upper and lower terminal plates of the vertebrae, the vertebra was considered square or rectangular. If four square/rectangular vertebrae were present, LSTV was identified as sacralization, and if six square/rectangular vertebrae were present, it was identified as lumbarization. The LSTV was classified using the Castellvi system, and the upper and lower terminal plates of L5 and S1 were also measured. The study concluded that no MRI parameter can precisely indicate the number of vertebrae without counting the levels, and these parameters may only be suggestive of LSTV rather than indicating the correct level. Building on this anatomical theme, Garg et al. [34] conducted a study in which they retrospectively evaluated 260 patients with a confirmed L5 level on MRI, analyzing anatomical landmarks such as the iliolumbar ligament, costal facets, aortic bifurcation, psoas origin, and conus medullaris. The sensitivity of each landmark for identifying L5 was 85%, 78%, 65.8%, 51.2%, and 58.5%, respectively, with specificities of 100%, 97.3%, 82.2%, 58.0%, and 68.0%. They concluded that, although some structures such as the iliolumbar ligament can help orientation, these landmarks are not sufficiently reliable for consistent numbering of L5 in cases of LSTV. Likewise, Chalian et al. [52] conducted a retrospective study involving 100 patients, 50 with LSTV and 50 healthy controls, and proposed two sagittal angle measurements (A and B) that may alert radiologists to the presence of transitional anatomy. Increased angles were associated with LSTV, suggesting their potential as screening signs.

The role of the iliolumbar ligament in LSTV identification was further explored in the article described by Hughes et al. [53], a retrospective study which reviewed 500 MRIs from patients presenting with lumbar pain, lumbar radiculopathy, or both. The study aimed to determine whether identifying iliolumbar ligaments is practically useful for numbering LSTV. Sixty‐seven patients were diagnosed with LSTV using this imaging method. The study concluded that the iliolumbar ligament is easily identifiable on lumbar spine MRI and always arises from L5. The authors suggested that its position can be used to safely assign lumbar levels in patients with LSTV. A similar approach was used by the retrospective study presented by Özbalci [47], which evaluated 1020 MRI images, and 114 patients were diagnosed with LSTV. The study diagnosed LSTV using MRI by identifying the iliolumbar ligament to define L5. They then classified LSTV according to Castellvi into patients with and without dysplasia of the transverse process. The study concluded that lumbosacral variations are common in patients with LBP and that these variations may be associated with degenerative and/or edematous changes that mimic sacroiliitis.

Some studies also focused on the clinical relevance of LSTV in the context of LBP and degenerative changes. Bhagchandani et al. [25] conducted a cross‐sectional study with 2016 patients, dividing them into 1009 with LBP and 1007 with radicular pain; of these, 149 and 174 patients had LSTV, respectively. In this cohort, MRI with T1 and T2 sequences was used. The study concluded that patients with sacralization represented > 80% of patients with LSTV and lumbar pain or radiculopathy, which may be explained by the high incidence of disc degeneration in the three levels immediately proximal to a sacralized vertebra, as well as a high incidence of terminal plate degeneration and facet joint tropism. The authors highlighted the strong association between LSTV and degenerative spine changes, particularly in patients with LBP. In a similar vein, Hanhivaara et al. [21] conducted a cross‐sectional study involving the 1966 northern Finland birth cohort. Of the 1468 cases, 310 were diagnosed with LSTV. Degenerative findings, including facet degeneration, disc protrusion, and Modic type I changes, were more frequent above the transitional segment (L3–L4 and L4–L5). The authors concluded that Castellvi type III is most strongly associated with LBP, supporting the biomechanical model of adjacent‐segment overload.

Finally, the association between LSTV and inflammatory LBP was highlighted by Türk et al. [41] who conducted a retrospective study in which 614 MRI images were reviewed, with 81 patients diagnosed with LSTV. The objective of the study was to assess the presence of LSTV in patients who underwent MRI due to suspected sacroiliitis. The study concluded that LSTV may present with LBP and should be considered in patients clinically suspected of having sacroiliitis. MRI was again noted as a useful modality for identifying coexisting pathologies.

### 3.5 Lumbar Spine X‐Ray (AP, Lateral, and Special Projections)

Six studies were identified that diagnosed LSTV using X‐ray, published between 2015 and 2024. These included one prospective study [5], two cross‐sectional studies [20, 22] and three retrospective studies [30, 31, 49].

Ravikanth and Mahumdar [5] conducted a prospective study that included 500 patients with inclusion criteria of only LBP, of which 134 were classified as positive for sacralization. This study aimed to classify anatomical variations in LSTV and determine, through simple X‐ray, if there is a relationship between sacralization and LBP. Of the total patients, the most common anatomical variant was Castellvi Type IA (7.6%), followed by Type IB (6.0%), Type IIA (1.8%), Type IIB (2.0%), Type IIIA (1.6%), Type IIIB (3.8%), and Type IV (0.8%). Patients with LBP and no malformation showed an average pain level of 2.2 compared to 5.2 in patients with LBP and a transitional vertebra. Similarly focused on LBP and hip pathology, Verhaegen et al. [20] conducted a cross‐sectional study, which included 153 patients, of which 19 had LSTV, with the aim of determining the prevalence of LSTV in young patients with hip pain compared to an asymptomatic volunteer group, the effect of LSTV on static and dynamic spinopelvic characteristics and evaluating the presence of LBP among young adult patients with hip pain and LSTV. In this study, the diagnosis of LSTV was made using an AP pelvic X‐ray. The Castellvi classification system was used to classify LSTV based on the degree of unilateral or bilateral articulation between the transverse processes of L5 and the sacrum. Spinopelvic parameters were measured in standing and sitting positions. This study concluded that LSTV was found in 8.5% of young adults, with no differences between patients with hip pathology and controls; and that individuals with LSTV have greater lumbar lordosis in standing, with altered mechanics at the adjacent cephalic level, which may predispose these individuals to degenerative changes at this level.

Sun et al. [22] conducted a cross‐sectional study with the aim of testing the hypothesis that there is a higher frequency of radiological anomalies in the spine in patients with acetabular dysplasia and evaluating the relationship between the radiological severity of acetabular dysplasia and the frequency of spinal anomalies. This study included 122 patients who presented hip pain and a final diagnosis of acetabular dysplasia and had an LSTV frequency of 39%–43%. The diagnosis and classification of LSTV were made using X‐rays. It was concluded that patients with acetabular dysplasia have a higher frequency of spinal anomalies observed in standard hip X‐rays.

Expanding on biomechanical parameters, in the study carried out by Benlidayi et al. [31], X‐rays were also used for the diagnosis of LSTV, and classification was performed according to Castellvi. Of 1588 patients evaluated retrospectively, 96 patients with Castellvi II or higher were included. The sacral tilt angle was measured on lateral X‐rays for comparison between groups. They concluded that the sacral tilt angle is significantly lower in patients with LSTV than in patients without LSTV, and that the sacral tilt angle does not differ between the types of LSTV. This may be useful for considering or confirming LSTV in patients with a smaller ST angle. Complementing this anatomical and diagnostic focus, Chiu et al. [49] conducted a retrospective study, which included the analysis of 998 X‐rays with the goal of investigating the variation in the number of thoracic and lumbar vertebrae, the prevalence of LSTV, and the prevalence of cervical ribs among surgical patients with adolescent idiopathic scoliosis. In this study, X‐rays were used to diagnose LSTV. They specified that vertebral numbering was performed starting from the C2 vertebra by identifying the odontoid process and continuing caudally to avoid omitting the cervical rib. All vertebrae with costal insertions (except cervical ribs), including full or unilateral ribs, were counted as thoracic vertebrae, and lumbar vertebrae were identified as vertebrae with no costal insertion after the most caudal thoracic vertebra. LSTV was defined as a lumbar vertebra in which one or both transverse processes were fused to the sacrum, through incomplete or complete bony fusion or a diarthrodial joint. This included sacralization of the lowest lumbar vertebral body and lumbarization of the highest sacral segment. In this study, seven different variations were identified in the number of cervical, thoracic, and lumbar vertebrae. The total prevalence of patients with atypical vertebral variation was 15.5%, and LSTV was found in 25.1% of the cohort. Furthermore, they stated that it is important to determine atypical vertebral variations rather than the absolute number of vertebrae. Therefore, it was concluded that due to differences in the number of morphologically thoracic and lumbar vertebrae, there may still be a risk of inaccurate identification.

Finally, in the study presented by Ani et al. [30], a retrospective analysis of 1548 patients was performed, of which 176 were included with LSTV. The aim was to determine whether patients with spinal deformity with sacralization of L5 should have PI and other spinopelvic parameters measured from the L5 terminal plate or S1. The diagnosis of LSTV was made using baseline full body standing X‐rays. The results showed that when measured using the upper terminal plate of L5, pelvic parameters were significantly smaller than those measured in relation to S1. They concluded that measuring PI and spinopelvic parameters in L5 with sacralized anatomy results in an underestimation of spinal deformity. Therefore, they suggest that surgeons may consider measuring PI and spinopelvic parameters in relation to S1 instead of L5 in patients with sacralized L5.

### 3.6 EOS 3D

Four studies were identified that diagnosed LSTV using EOS 3D. These articles were published between 2016 and 2022. Of these, one study was prospective [15] and three retrospective studies [36, 40, 50].

Okamoto et al. [36] conducted a retrospective study with 291 healthy adult patients with no history of spine disease, using biplanar full‐body EOS imaging to evaluate the prevalence of LSTV and its influence on global sagittal alignment. They identified 14 patients with 23 vertebrae (sacralization) and 16 with 25 vertebrae (lumbarization), for an overall prevalence of 10.3%. Compared with normal spines, the sacral base was located higher in the L4 group (sacralization) and lower in the L6 group (lumbarization). Participants with LSTV showed significant differences in global alignment, with greater cervical lordosis (C2–C7) and increased lumbar lordosis in both L4 and L6 groups. Conversely, other spinopelvic parameters were reduced in L4 and increased in L6. On the basis of the analysis of the results, the authors proposed radiographic principles for vertebral numbering based on rib presence, intervertebral disc continuity, and sacral base identification, improving consistency in LSTV assessment. The authors concluded that the spinopelvic parameters of the LSTV population significantly differed from those of the normal spine population due to differences in the location of the sacral base; thus, they proposed an accurate method for numbering the vertebrae using coronal and sagittal full‐body images.

In the article performed by Becker et al. [40], a retrospective study was conducted with 51 patients with L5/S1 osteochondrosis to investigate lumbar spine mobility and the distribution of segmental movement in patients with LSTV. In this study, full spine radiographs obtained from EOS were used to diagnose LSTV and classify it according to Castellvi, following parameters for vertebral numbering and spinopelvic measurements. The authors mention that the number of lumbar vertebral bodies was classified by counting caudally from C1 in full‐spine images. For the cervical spine, 7 vertebrae were assumed, and 12 for the thoracic spine. L1 was defined as the 20th vertebra, and lumbar vertebrae were assumed if there were 25 vertebrae with at least rudimentary discs between them. On the basis of the results, the authors concluded that LSTV has a significant effect on lumbar spine movement patterns with the use of flexion‐extension radiographs. This is because reduced movement was demonstrated in the transitional segment, as well as an increased proportion of mobility in the cranial adjacent segment. Likewise, Zhou et al. [50] retrospectively evaluated 1869 radiographs and identified 70 patients with transitional lumbosacral vertebrae (3.7%). Among these, 82.9% had lumbarized sacral segments and 17.1% had sacralized lumbar segments. The study demonstrated that choosing different sacral endplates (cephalad vs. caudal) for measurements significantly alters pelvic and global alignment parameters. When the caudal transitional segment was selected, mean pelvic incidence, pelvic tilt, and sacral slope were markedly higher (PI: 66.8° vs. 44.3°; PT: 25.1° vs. 12.7°; SS: 41.6° vs. 31.6°, *p* < 0.001). Similarly, lumbar lordosis and global parameters such as sagittal vertical axis and T1–pelvic angle also increased significantly. Therefore, they mentioned that surgical errors are more likely when lumbar MRI is reported without additional images; as the use of S1–S2 disc morphology, vertebral body shape, and the lumbosacral angle to define vertebral levels leads to the erroneous identification of LSTV and spinal levels.

Price et al. [15] conducted a study based on prospective full‐body EOS spine databases, diagnosing LSTV in 11 patients. This study aimed to provide normative values for spinopelvic measures in S1 lumbarization and to investigate correlations between lumbar lordosis and PI, measured at the first immobile sacral vertebra, S2, and the incidence of L6 in the true S1. Regarding radiological parameters, the authors also followed principles for vertebral numbering. Patients with S1 lumbarization were identified based on the number of lumbar vertebral bodies counted from the cervical spine caudally, assuming seven cervical vertebrae and 12 thoracic vertebrae. Patients were positioned in a standardized standing posture to ensure reproducible spinopelvic measurements. On the basis of the results, it was concluded that incomplete spine images could lead to an erroneous estimate of lumbarization prevalence, and that patients with lumbarization have higher lordosis values, with lordosis now being estimable during preoperative planning for this group.

### 3.7 Mixed

Thirteen articles were identified in which the diagnosis of LSTV was made using a combination of diagnostic techniques. These articles were published between 2010 and 2024. Among these, there were two literature reviews [4, 12], two prospective studies [13, 14], three cross‐sectional studies [18, 19, 24] and six retrospective studies [29, 37, 38, 39, 44, 51].

Two literature reviews focused on vertebral numbering and the clinical relevance of imaging findings in LSTV. Lian et al. [12] reviewed current literature on precise methods for vertebral numbering. In this study, the diagnosis of LSTV was evidenced through various radiological techniques. Their results showed that the gold standard for spinal segment numbering in patients with LSTV remains the acquisition of full‐spine images and caudal numbering starting from C2. If unavailable, the use of the iliac crest tangent sign in coronal MRI has a reliable sensitivity and specificity for accurate LSTV numbering, with a sensitivity and specificity of 81% and 64%–88%, respectively. The role of other anatomical markers such as the right renal artery, superior mesenteric artery, aortic bifurcation, and the conus medullaris in identifying vertebral levels is unreliable and should not be used. In conclusion, the authors highlighted that full‐spine MRI should be considered when transitional anatomy is suspected, as numerical variants are common and may coexist with thoracic or cervical anomalies. Similarly, Konin et al. [4] conducted a literature review aimed at addressing issues related to correctly identifying and numbering LSTV, as well as detecting imaging findings related to lumbar pain genesis. Several diagnostic techniques were used in this study. The results indicated that without high‐quality full‐spine images, no infallible method exists for accurately numbering a transitional segment. Therefore, identification, communication with the referring physician, and correlation of intraoperative and preoperative images become extremely important. The study concluded that LSTV are common spinal anomalies that require accurate identification and numbering of the affected segment. A better understanding of the biomechanical alterations caused by LSTV can help radiologists recognize and interpret imaging findings in patients with lumbar pain and a transitional segment.

Two prospective studies combined multiple imaging modalities, including radiographs and MRI, for accurate LSTV diagnosis. Sencan et al. [13] examined 64 patients with radicular LBP undergoing lumbar transforaminal epidural steroid injection. Sacralization was identified by MRI and classified radiographically by Castellvi type. Pain and disability scores improved significantly in all patients (*p* < 0.05), but treatment success at 3 months was lower in those with sacralization (44.8%) compared with patients without LSTV (65.6%, *p* = 0.026). The authors concluded that sacralization may reduce therapeutic response and should be considered when evaluating treatment outcomes. In a related prospective study, Omidi et al. [14] conducted a study in which lumbar sacralization was diagnosed using radiological images, including oblique and lateral views of the spine, radiographs, and T1 and T2 weighted MRI images. Both imaging techniques were required for numbering and LSTV diagnosis. LSTV categorization was reported according to Castellvi's classification system, and the authors reported that transitional anatomy was associated with alterations in pelvic incidence and lumbar curvature, suggesting that combined imaging improves diagnostic accuracy and preoperative planning.

Among the cross‐sectional studies, three focused on combining radiography, MRI, or CT to evaluate LSTV. Shaikh et al. [19] performed a cross‐sectional study aimed at determining the frequency of LSTV in patients with LBP and the role of the L5 iliolumbar ligament origin in LSTV cases. This study evaluated both radiographs and MRIs together to determine the presence of LSTV. Initially, simple radiographs were reviewed, followed by MRI scans. LSTVs were classified according to Castellvi. LSTV was identified in 18% of patients, and in 44% of those cases, the iliolumbar ligament originated anomalously. The study concluded that LSTV occurs frequently in patients with lumbar pain. Furthermore, in the presence of LSTV, the iliolumbar ligament is not a reliable marker for identifying L5. In another cross‐sectional study, Tatara et al. [18] examined 56 patients with the goal of determining the optimal vertebral level for LSTV to measure PI and pelvic tilt. They concluded that if the measured PI is lower than the reference range, it is likely that PI was measured with LSTV as S1, and for this reason, it would be better to remeasure the PI with LSTV as the lowest lumbar vertebra. Conversely, if the measured pelvic tilt is higher than the reference range despite a high PI and no sagittal imbalance, it is likely that pelvic tilt was measured with LSTV as the lowest lumbar vertebra (*p* < 0.05). Furthermore, the study authored by Hanhivaara et al. [24] was a cross‐sectional study that included 852 patients undergoing lumbar imaging studies using all three modalities, initially evaluated for LSTV presence through CT scans, although all three modalities were employed. This study aimed to compare the diagnostic performance of conventional radiography, CT, and MRI. In total, 100 patients with LSTV anatomy were identified. The results reported superior diagnostic efficacy for CT: the sensitivity, specificity, accuracy, and balanced accuracy were 76%, 93%, 77%, and 84%, respectively. For MRI, the metrics were 54%, 88%, 56%, and 68%, and for radiography 32%, 85%, 42%, and 59%, respectively. CT also demonstrated good inter‐reader reliability (*κ* = 0.63–0.71), while MRI and radiography showed only fair agreement (*κ* = 0.24–0.56 and *κ* = 0.16–0.32, respectively). The conclusion was that CT provided the highest diagnostic performance in all metrics with good reliability among readers. Additionally, MRI and conventional radiography showed poor sensitivity and accuracy and should therefore be interpreted with caution when classifying LSTV.

A group of retrospective studies also relied on mixed modalities for diagnosis and classification of LSTV. Farshad‐Amacker et al. [37] conducted a retrospective study with 155 subjects. The objective was to evaluate inter‐reader reliability for LSTV detection and classification using standard AP radiographs and report its accuracy using intermodality statistics in comparison to MRI as the gold standard. The authors consider that radiographs are insufficient for LSTV diagnosis. Therefore, their study used both radiographs and MRI to identify and classify LSTV according to Castellvi. Agreement between modalities was poor (*κ* = 0.29), supporting the need for combined imaging to improve diagnostic accuracy. In a related retrospective study, Peckham et al. [38] used both radiographs and MRI to count vertebrae and classify LSTV. First, vertebral numbering considered the first seven vertebrae as cervical and the following 12 as thoracic, even in cases with an abnormal number of ribs. In cases with 13 rib‐bearing vertebrae, it was considered “lumbarization” with L1 having supernumerary ribs. After T12, vertebrae were counted as lumbar, extending to the lumbosacral junction. Vertebra 24 was considered L5 in all cases. LSTV classification considered whether lumbar transverse processes had unilateral or bilateral un‐fused joints with the sacrum (partial sacralization of L5), classifying them as Castellvi 1 or 2. If transverse processes were fused unilaterally or bilaterally with the sacrum (complete sacralization of L5), LSTV was classified as Castellvi 3 or 4. The authors emphasized that numbering errors were common when rib variants were present, underscoring the importance of full‐spine imaging for accurate labeling. Furthermore, Hou et al. [39] also conducted a retrospective study in which LSTV was initially diagnosed using radiographs and confirmed with CT. Both imaging techniques were used for LSTV diagnosis. Simple lumbar AP radiographs were evaluated for LSTV morphological abnormalities (types II–IV according to Castellvi classification). The patient was then examined with spiral CT scans from T12 to the sacrum for LSTV confirmation. The results showed that 35.2% of suspected LSTV types assessed by AP radiographs were inconsistent with the final types assessed by CT. The article concluded that radiographs could detect LSTV correctly but could not provide a precise type of classification, while CT provided detailed information between the pelvic tilt and sacral area and could be considered the gold standard for detecting and classifying LSTV.

Additionally, Tatara et al. [29] conducted a retrospective study where LSTV was detected in 3D CT images, and vertebral numbering was done with spine radiographs and classified according to Castellvi. For presacral vertebrae, numbering was done caudally from C2 using full‐spine radiographs. The lowest lumbar vertebra was identified as lumbar when above the iliac arch line, as normal lumbar vertebrae are located above that line. Additionally, the number of vertebrae and sacral foramina was identified when below the promontory in the CT images. The authors highlighted that correct identification of the lowest lumbar vertebra above the iliac arch line reduces misclassification and improves preoperative accuracy in surgical planning. Additionally, Carrino et al. [51] performed a retrospective review of 147 subjects using spine radiographs as the reference standard to determine total and segmental vertebral count and transitional anatomy. LSTV diagnosis was made with MRI and radiographs. They reported that anomalous total vertebral counts occurred in 8.2% of patients, and that the ligament was identifiable in 85.7% of cases, being located at L5 in 96.8%, though unreliable in anomalous numbering. The study concluded that iliolumbar ligament denotes the lowest lumbar vertebra, which does not always represent L5. Additionally, a well‐formed, complete S1–2 intervertebral disc is associated with LSTV, and LSTV is associated with abnormal vertebral numbering.

Lastly, Byvaltsev et al. [44] conducted a retrospective study. LSTV was detected using MRI, CT, and radiographs. To assess the type of lumbosacral junction anomaly in the study patients, detailed examinations of their lumbar radiographs, MRIs, and CT scans were performed. The number of vertebrae was counted from the cranio‐cervical junction according to radiographic data. The Castellvi classification was applied with a simplified modification grouping the types into three functional categories: enlarged transverse process (types I), pseudoarthrosis (types II), and fused (types III–IV). The authors found that the fused group was the most frequent and correlated with decreased segmental mobility and lumbar pain, reinforcing its biomechanical significance.

## 4 Discussion

Historically, opinions on the clinical impact of LSTV have varied widely, from early interpretations as anthropological curiosities to later recognition as a cause of mechanical low back pain. These contrasting views underscore the need for standardized imaging‐based classification and clinical correlation.

In 1981, Wigh's review of 200 positive myelograms revealed no cases of hernia at the LSTV level. However, the variability of findings in different studies led Castellvi and colleagues to investigate the potential association between nucleus pulposus herniation and LSTV by conducting their own study [3]. This study led to the Castellvi classification to organize the types of LSTV. In 1984, Castellvi et al. [3] conducted a retrospective study that included 200 consecutive cases from 1979 with myelographic evidence of nucleus pulposus herniation. The myelograms were initially interpreted by neuroradiology staff and later reviewed independently by one of the authors. From this study, after their own classification and correlation with the myelographic findings of herniated nucleus pulposus, they concluded that type I represents a subtle manifestation of LSTV, with no discernible difference in the appearance of the hernia localization. In types III and IV, no hernias were observed at the LSTV level, nor was there a high incidence of hernias immediately proximal to it. However, the most notable finding was the type II anomaly, which showed a higher prevalence of disc herniation just above the LSTV and presented nucleus pulposus herniation at the transitional level.

The Castellvi classification system represented an initial effort to categorize LSTV, contributing to increasing awareness of this frequently overlooked anatomical variation. Among the advantages of its creation are the promotion of coherence and the standardization of terminology and diagnostic criteria, facilitating communication between neurosurgeons, neuroradiologists, orthopedists, and other healthcare professionals and researchers. Additionally, it offers insight into the natural history of pathologies related to the presence of LSTV, helping in patient counseling and medical decision‐making. However, the Castellvi classification has its limitations. It lacks the ability to predict the need for treatment, as it does not consider certain variables now recognized as significant in Jenkins' classification. Furthermore, the absence of studies linking this classification system to symptoms may limit its clinical usefulness. These deficiencies could increase the risk of misdiagnosis and inappropriate treatment of LSTV‐related conditions [55].

**FIGURE 3:**
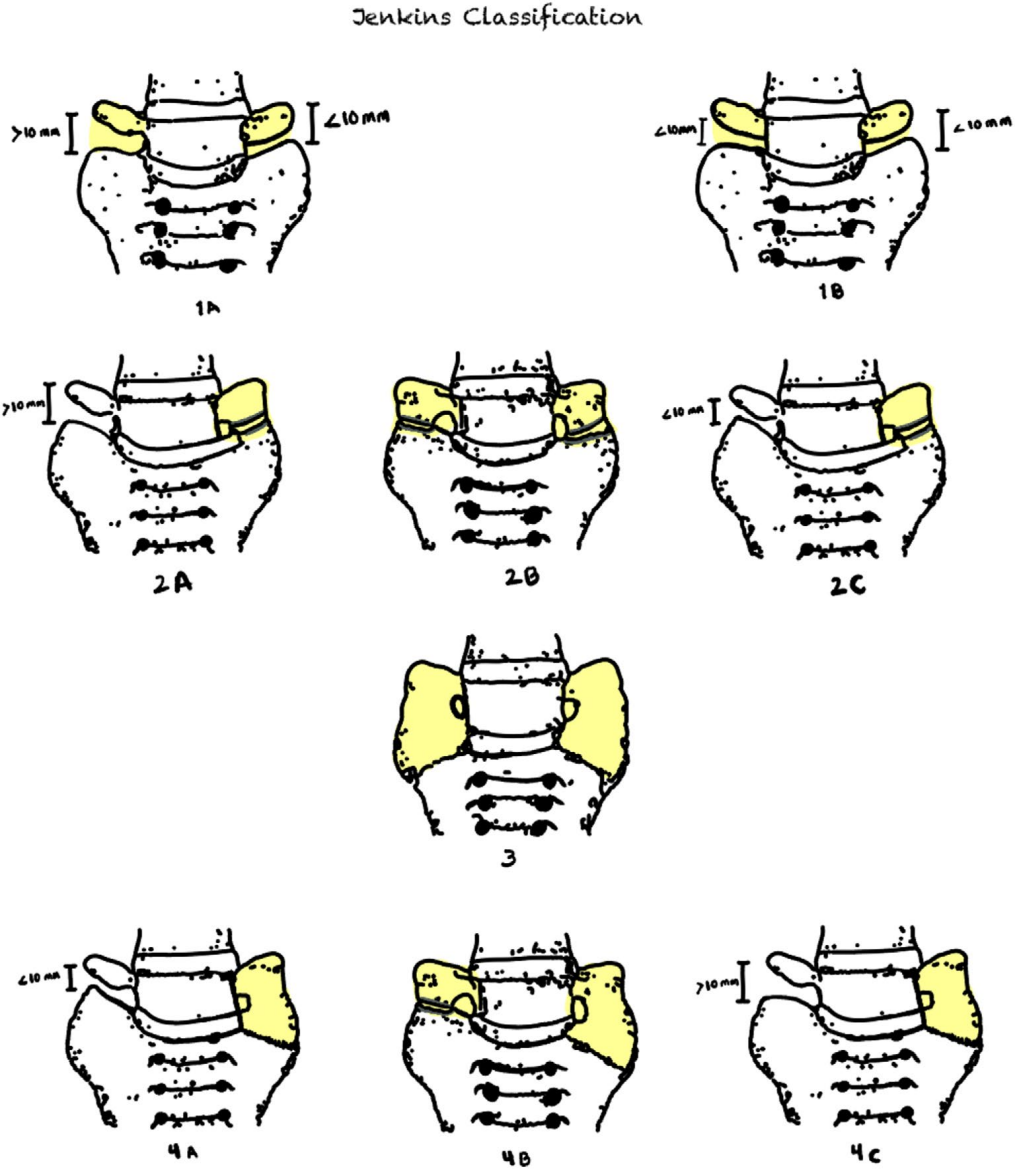
Graphic illustration of Jenkins classification. Four categories with nine subcategories.

The Castellvi classification has been accepted since its creation. Despite this, Jenkins et al. [55] proposed complementing the Castellvi classification. They conducted a retrospective cohort study of 150 new patients for the treatment of back, hip, groin, and leg pain using MRI, CT, and radiography. From this study, they discovered that the Castellvi classification excludes two types of anatomical variants: the prominent anatomical side and the possible contact of the transverse process and the iliac crest 56. Therefore, their aim was to introduce and validate a new classification system, the “Jenkins classification” for LSTV (Figure 3).

The diagnosis of LSTV is of vital clinical importance due to the multiple implications that this anatomical variation can have on the health and treatment of patients. LSTV is frequently associated with LBP, known as Bertolotti's syndrome. This pain may arise from intrinsic inflammatory processes, biomechanical alterations in the spine, or degenerative changes in the discs and facet joints, particularly in certain types of LSTV according to the Castellvi classification. Its impact is not limited to pain, as it may also influence spinopelvic alignment parameters, essential for evaluating sagittal balance and global spinal stability, which are crucial in patients with deformities or degenerative conditions. From a surgical perspective, accurate identification and proper numbering of the vertebrae are essential to avoid errors in procedures such as spinal fusions or epidural injections, where incorrect localization may compromise treatment effectiveness and lead to complications. Additionally, the presence of LSTV requires adjustments in standard evaluation protocols, designed under the assumption of five lumbar vertebrae, to ensure accurate results in therapeutic planning. Beyond these diagnostic and planning considerations, the clinical relevance of LSTV, particularly sacralization, also extends to its potential biomechanical consequences. Some findings indicate that sacralization significantly increases the risk of higher‐grade spondylolisthesis, especially at the L4–L5 level, due to the altered mechanical stress distribution [56, 57]. The fusion of L5 to the sacrum reduces motion at the lumbosacral junction, consequently placing greater strain on the adjacent superior segment. This biomechanical imbalance predisposes the L4–L5 level to degeneration, vertebral slip, and even motor deficits [57]. Such degenerative changes and instability have direct clinical implications, potentially necessitating earlier or more aggressive intervention. Therefore, recognizing and reporting LSTV is crucial not only for accurate diagnosis but also for anticipating the progression of spinal pathologies and tailoring individualized treatment strategies. However, the lack of standardization in radiological findings and difficulties in properly classifying this anomaly complicate its diagnosis, underscoring the need for a more uniform and precise diagnostic approach to optimize clinical evaluation, guide therapeutic decisions, and improve patient outcomes.

This study demonstrates the different techniques for diagnosing LSTV. Some studies agree with the parameters to consider for its diagnosis, while others vary. It is important to highlight the radiological findings used to arrive at the correct Castellvi classification, and this is discussed considering the type of diagnostic technique and the form or parameters followed. The results of this article suggest analyzing the appropriate measurement level and stable spinopelvic parameters, with the aim of facilitating understanding of the radiological variability in the evaluation and diagnosis of individuals with LSTV, along with its proper staging according to the Castellvi classification.

Usmani et al. [9, 10] described several cases and radiological findings using positron emission tomography (PET‐CT) and sodium fluoride‐18F bone CT to stage LSTV using the Castellvi classification. These cases were:

- *LSTV IIa*: NaF images show an increased tracer uptake in the pelvis, corresponding to the broad transverse process of the L5 vertebra, which forms a diarthrodial joint with the sacrum (hemisacralized transverse process of L5). The volumetric image shows tracer uptake in the diarthrodial joint. Another case shows that the transaxial and coronal images extend the transverse process of the L5 vertebra, forming a diarthrodial joint with the sacrum, with increased tracer uptake in the fused PET‐CT images. The findings are consistent with osteoblastic activity in the hemisacralized transverse process of L5.
- *LSTV IIb*: CT images show the broad bilateral transverse processes of the L5 vertebra forming a diarthrodial joint with the sacrum, without increased tracer uptake in the fused PET/CT images in the hemisacralized transverse process of L5.
- *LSTV IIIa*: The CT image shows complete fusion of the transverse process on one side with the sacrum, without the corresponding uptake in the fused images.

Iplikcioglu and Karabg [21, 27, 28] use CT for the diagnosis of LSTV and described that to define the Castellvi classification grade of LSTV. Instead, Hanhivaara et al. [42] described certain subtypes not included in the original Castellvi classification: LSTV type IIa and IIIa with contralateral enlarged transverse processes. Therefore, it is proposed that these new subtypes IIc and IIIc be consistent additions to the Castellvi classification.

Several studies showed the relationship between lumbopelvic parameters in patients with vertebral anomalies or LSTV. In the study by Desai et al.35, lumbopelvic parameters were measured, and they concluded that patients with Bertolotti's syndrome had a significantly higher PI and were more likely to present adjacent‐segment disease compared to control patients (without LSTV). However, after controlling for some variables, no significant association seemed to exist within the cohort of patients with Bertolotti.

On the other hand, Kassir et al. [11] reported a case in which bone scintigraphy with 99m Tc‐methylendiphosphonate was requested, and the planar images showed a focus of increased tracer activity in the lumbosacral area on one side over the same‐side sacroiliac joint. Therefore, a PET‐CT was requested, which showed a focus of increased tracer activity in a transverse process on the same side, consistent with the scintigraphy findings. They mention that, although the presence of a transitional vertebra can be established with simple radiography, CT, or MRI, the demonstration of stress on the transverse‐sacral joint is best achieved using bone scintigraphy with SPECT.

Regarding diagnostic techniques, Ravikanth and mahumdar [5] mention that LSTVs have traditionally been identified through lateral radiographs and Ferguson's views. These “squares” and “wedges” represent a spectrum of morphological changes of the vertebral body and cannot be reliably used to definitively identify an LSTV. Not only is the identification of an LSTV important, but the precise numerical identification of the vertebral segments on MRI is essential before surgery. Similarly, they state that it is important to correlate MRI with intraoperative radiographs to confirm the disc level during surgery. This is because it can often be problematic to determine whether an LSTV is an S1 lumbar or an L5 sacralized vertebra using MRI alone. This can lead to surgical errors when a lumbar spine MRI is reported without accompanying conventional radiographs or MRI localizers. The MRI correlation with other images is supported by Lian et al. [12], who mention that surgical errors are more likely when lumbar MRI is reported without additional images; as the use of S1–S2 disc morphology, vertebral body shape, and lumbosacral angle to define vertebral levels leads to incorrect identification of LSTV and correct spinal levels.

Overall, our review shows that the diagnostic performance of imaging modalities varies, and each has a distinct role in clinical practice. Radiography remains useful for initial vertebral numbering but lacks accuracy for classification. CT provides the highest sensitivity and specificity and should be considered the gold standard for definitive diagnosis and preoperative planning. MRI is valuable for assessing the iliolumbar ligament and coexisting soft‐tissue pathologies, though it is limited for accurate vertebral numbering. EOS offers unique advantages for whole‐spine evaluation and sagittal alignment, but its availability is restricted. Nuclear medicine techniques such as PET‐CT and bone scintigraphy have a niche role in detecting active bone remodeling in symptomatic cases. Taken together, these findings imply that imaging choice should be tailored to the clinical context: simple numbering in incidental LSTV, but comprehensive methodology when surgical planning, interventional procedures, or biomechanical assessments are required. This tailored approach can reduce diagnostic errors, prevent wrong‐level surgery, and improve patient outcomes.

Taking into account the discussion and the literature review, our proposal for diagnosis and classification of LSTV is:

1. Initial Identification Using Diagnostic Imaging

Perform craniocaudal AP radiographs of the entire spine from C2 downward. Evaluate morphological anomalies and distinguish hypoplastic ribs from lumbar transverse processes. This allows for determining the number of thoracic segments and correctly assigning vertebral numbering. Consider imaging the entire spine as the most accurate method for numbering the vertebrae, detecting cervical, thoracic, or transitional anomalies that may be present. Define L1 and S1 as vertebrae 20 and 25 only if there are 7 cervical, 12 thoracic, and 5 lumbar vertebrae. Standard AP radiographs alone are insufficient for detecting or classifying LSTV. They should be supplemented with other imaging techniques.

2 Confirmation and Classification with Computed Tomography (CT)

We consider CT to be and should remain the gold standard for the diagnosis of LSTV, as it offers the best diagnostic performance and high reliability between readers. It is especially useful for classifying LSTV based on morphology and anatomical variants. Evaluate spinopelvic parameters such as PI, pelvic tilt, lumbar lordosis, using the terminal plate closest to the normative average PI value as a reference. If the measured PI is outside the normative range, review and adjust the measurements, considering the LSTV as the lowest lumbar vertebra or the highest sacral vertebra, as appropriate

3 Use of Magnetic Resonance Imaging (MRI) in Specific Cases

Perform MRI of the lumbar spine to identify iliolumbar ligaments, which commonly emerge from the transverse processes of L5. Their position can be used to assign lumbar levels in patients with LSTV. In patients with LSTV, if the iliolumbar ligament is not identified at the superior level, the LSTV may be considered L5. If the ligament arises above the LSTV, it is numbered as S1. Use axial T1 and T2‐weighted images to observe the iliolumbar ligament as a low‐signal intensity structure.

4 Morphological Analysis for Classification and Surgical Planning

Identify key morphological features such as hypoplastic or absent facet joints in the L5‐S1 fusion and facet joints between S1‐S2 in cases of lumbarization. Use the Jenkins LSTV classification for typification and therefore for its respective use in surgical planning.

5 Functional Evaluation and Biomechanical Effects

Determine the influence of LSTV on the biomechanics of the spine, evaluating its impact on lumbar muscle degeneration and reduced muscle volume.

6 Additional Examinations in Complex Cases

If there are anomalous segmentations along with LSTV, identifying the iliolumbar ligament may not be enough. In these cases, consider the possibility of performing additional comprehensive examinations with MRI or CT for a more accurate assessment.

Our proposed diagnostic methodology is intended for radiologists, neurosurgeons, spine surgeons, and interventional pain specialists in cases where precise vertebral level identification is critical for procedures (e.g., spinal fusions, epidural injections, sagittal balance analysis). It is not necessary for incidental, asymptomatic LSTV detected during imaging for unrelated reasons. Instead, it should be reserved for patients with symptomatic LSTV, those undergoing surgical planning, or cases where misidentification could result in procedural errors.

We believe that this approach allows for a comprehensive, accurate diagnosis and is useful for therapeutic planning, reducing errors, and improving clinical outcomes in patients with LSTV.

This study has some limitations, including an incomplete literature search, as other databases may contain articles with a high impact on the objective of this study. Additionally, many of the studies had incomplete information, as they only mentioned the diagnostic technique used to detect LSTV and did not specify how the diagnosis was made or which radiological findings were used. Furthermore, there may be selection bias as the search was limited to title and abstract, suggesting that some relevant articles may have been excluded.

Future research on LSTV should aim to develop a comprehensive meta‐analysis that synthesizes current evidence and supports the creation of standardized imaging protocols and diagnostic criteria, including the consistent application of Castellvi classification. Large‐scale, prospective, multicenter studies are needed to compare the diagnostic performance of CT, MRI, and radiography, using standardized vertebral numbering and detailed spinopelvic measurements. Incorporating artificial intelligence and machine learning into radiological assessment could reduce diagnostic errors and interobserver variability. Additionally, further investigation into the functional impact of different LSTV subtypes, particularly in interventional procedures and biomechanical evaluations, is warranted. Emerging imaging techniques like EOS should be evaluated for their utility in assessing sagittal alignment and spinopelvic parameters. Lastly, as anatomical variability continues to challenge existing classification systems, future studies should explore potential refinements to the Castellvi classification or integrate complementary systems such as Jenkins' to enhance clinical relevance and diagnostic precision. Therefore, this study shows the need for a meta‐analysis on this topic to reach a possible consensus for diagnosing LSTV and correctly using the Castellvi classification.

## 5 Conclusions

In conclusion, LSTV is a common condition, and many patients may have anatomical variants in the spine. This has led to varying literature regarding the most appropriate diagnostic technique and the aspects to consider. Each diagnostic technique should be evaluated, considering its sensitivity and specificity; this would provide clearer information for diagnosing LSTV, especially in patients with anatomical variants. When choosing the diagnostic technique to use, healthcare professionals should consider measuring spinopelvic parameters in these patients, identifying anatomical landmarks accurately, and ensuring that the image is taken in the correct position and manner. This would lead to a more accurate diagnosis and allow for a more secure classification using the Castellvi or Jenkins classification. Our proposed methodology for diagnosing LSTV is: first, using simple AP radiographs to perform craniocaudal vertebral numbering and assess morphological anomalies. Second, if there is suspicion, perform a CT as the gold standard for diagnosing LSTV, considering its high sensitivity and specificity; and measure spinopelvic parameters to correlate with LSTV. Third, use MRI in special cases. Fourth, perform a morphological analysis and utilize the Jenkins classification for LSTV classification.

## Author Contributions

Both authors confirm responsibility for the following: study conception and design, data collection, analysis and interpretation of results, and manuscript preparation. All authors reviewed the results and approved the final version of the manuscript.

## Funding

The authors have nothing to report.

## Disclosure

All figures included in this paper are the author's own work and have been created specifically for the purposes of this study. No copyrighted or third‐party material has been used in this study.

## Ethics Statement

The authors have nothing to report.

## Consent

This study did not require informed consent for participation, as there was no direct contact with patients. The research was conducted as a systematic review of previously published studies, all of which obtained informed consent from their respective participants. Since our analysis is based solely on publicly available data and does not involve new data collection or interaction with individuals, there are no ethical concerns or implications requiring additional consent.

## Conflicts of Interest

The authors declare no conflicts of interest.

## Data Availability

All data are publicly available through the databases used in the review and references are included in the manuscript.
